# Clampdown of inflammation in aging and anticancer therapies by limiting upregulation and activation of GPCR, CXCR4

**DOI:** 10.1038/s41514-018-0028-0

**Published:** 2018-08-30

**Authors:** Raji R. Nair, Shreyas V. Madiwale, Deepak Kumar Saini

**Affiliations:** 1 Department of Molecular Reproduction, Development and Genetics, Indian Institute of Science, Bangalore, India; 2 Centre for Biosystems Science and Engineering, Indian Institute of Science, Bangalore, India; 3 Present Address: Department of Cell and Developmental Biology, University of Michigan Medical School, Ann Arbor, MI USA

## Abstract

One of the major pathological outcomes of DNA damage during aging or anticancer therapy is enhanced inflammation. However, the underlying signaling mechanism that drives this is not well understood. Here, we show that in response to DNA damage, ubiquitously expressed GPCR, CXCR4 is upregulated through the ATM kinase-HIF1α dependent DNA damage response (DDR) signaling, and enhances inflammatory response when activated by its ligand, chemokine CXCL12. A pharmacologically active compound screen revealed that this increased inflammation is dependent on reduction in cAMP levels achieved through activation of Gαi through CXCR4 receptor and PDE4A. Through in vivo analysis in mice where DNA damage was induced by irradiation, we validated that CXCR4 is induced systemically after DNA damage and inhibition of its activity or its induction blocked inflammation as well as tissue injury. We thus report a unique DNA damage-linked inflammatory cascade, which is mediated by expression level changes in a GPCR and can be targeted to counteract inflammation during anticancer therapies as well as aging.

## Aging and Inflammation: Targeting a cellular receptor reduces the deleterious inflammation during aging and anti-cancer therapy

A sensing protein that is increased in response to DNA damage can be targeted to reduce inflammation and collateral damage during anti-cancer therapy and aging. Scientists at Saini Lab at the Indian Institute of Science have identified the protein that drives sustained and detrimental inflammation when the DNA of cells are damaged, such as during normal human aging or during anti-cancer therapy. Furthermore, blocking the functions of this protein and associated pathway was able to reduce the inflammation to less harmful levels. This discovery could potentially enable safer and more effective anti-cancer therapy by protecting non-cancerous cells surrounding tumors from lethal inflammation. Further studies on this protein could also reduce age associated inflammation, allowing us to age gracefully and healthily.

## Introduction

DNA damage in cells triggered through either intrinsic factors like oxidative and nitrosative stress or extrinsic factors like radiation, chemical agents etc. primarily leads to development of an inflammatory response, which is intricately tied with the cell fate decisions. Depending on the quantum of damage, either repair, senescence or death pathways are activated.^1–3^ While severe DNA damage is utilized to kill cancer cells as it triggers death, moderate but persistent damage leads to senescence, where cells enter an irreversible state of growth arrest, which is known to recapitulate as well as contribute to organismal ageing.^1,4,5^ It is now well established that one of the hallmarks of damaged as well as senescent cells is enhanced inflammation, which is mediated by DNA damage response (DDR).^6–9^ This inflammation facilitates homing of immune cells for clearing the dead or damaged cells. However, presence of chronic and unresolved inflammation is deleterious and is implicated as a major driver of disorders including cancer, loss of tissue function and deterioration in quality of life.^10,11^ In the present study, we aimed to identify molecular players, primarily GPCRs which regulate DDR dependent inflammation. Towards this, we used chemotherapeutic agent treatment, radiation exposure, and cellular senescence as models to activate DDR and study inflammatory response. Previous studies have identified molecules like p38 MAPK, NF-κB as regulators of inflammation in DDR^12,13^ but no clear role for GPCR signaling has been reported.

Previously, inhibition of CXCR2, receptor of chemokine CXCL8 (IL8), an inflammatory cytokine was reported to suppress senescence and cause premature senescence when ectopically overexpressed,^14^ hinting that this receptor might be regulating DDR. Similarly, another receptor CXCR4, was found to be upregulated in many cancers,^15^ aged neutrophils as well as in senescent cells.^16,17^ It has also been reported that elevated expression levels of CXCR4 receptor is generally an indication of increased metastatic potential of the cancer cells.^18^ Some recent studies have also targeted this receptor-ligand (CXCR4-CXCL12) axis to counteract therapy induced inflammation as well as metastasis, however the mechanism underlying this effect is still not clear.^15,19^ Here, we provide mechanistic and in vivo evidence of regulatory role of CXCR4 receptor in DDR. We show that CXCR4 expression is upregulated by DDR either during anticancer therapy or senescence through an ATM-kinase and HIF1α activation dependent pathway and the receptor upregulation and activation is specifically responsible for generating the enhanced inflammatory response by the damaged cells. The mapped molecular signaling cascade was conserved in both cellular as well as mouse model of radiation-mediated injury. Screening of pharmacologically active compound library backed the findings and identified many molecules that could be used for suppressing the DDR-dependent inflammation.

## Results

### CXCR4 expression is induced by DNA damage response

**Fig. 1:**
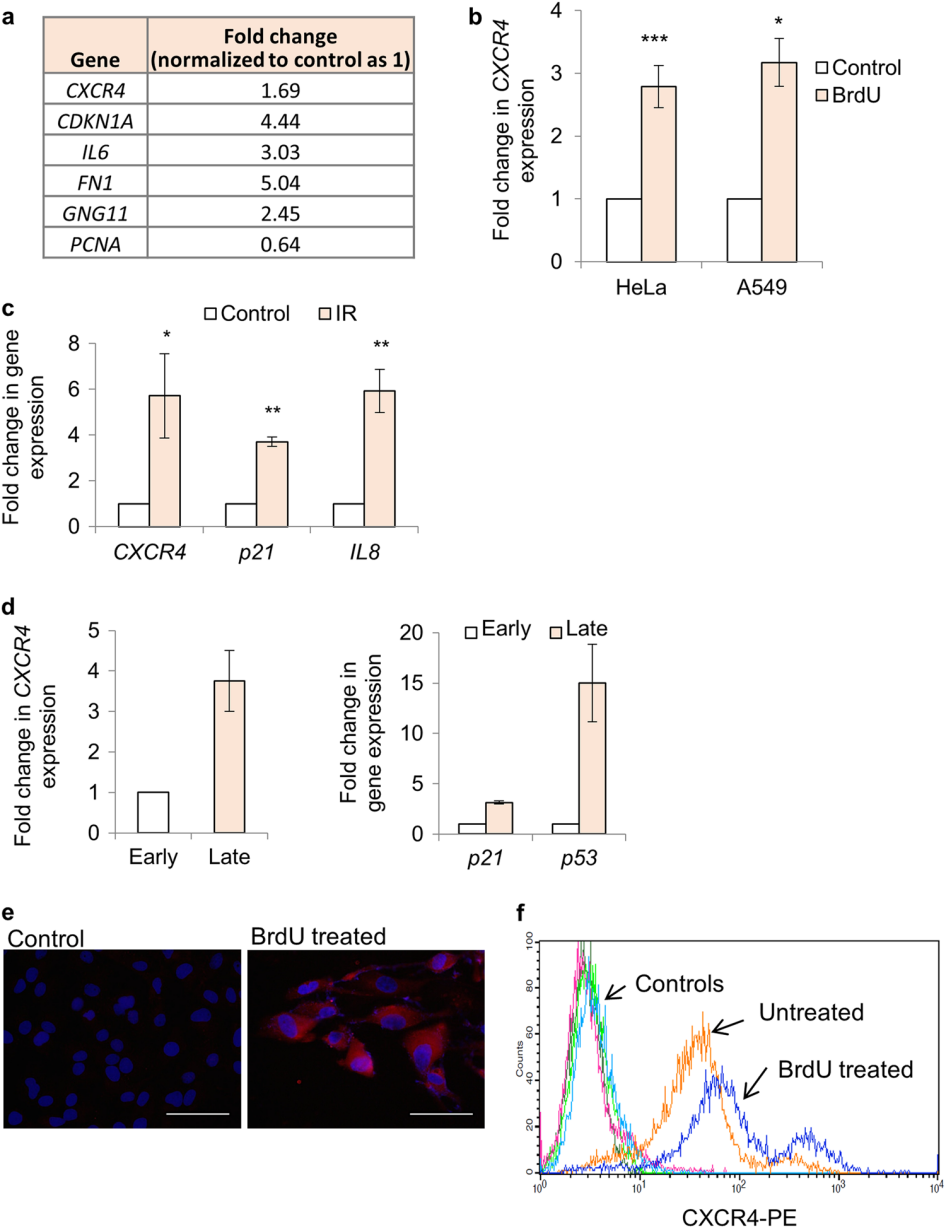
*CXCR4* expression analysis in response to DNA damage. **a** Gene expression changes after BrdU treatment in HeLa cells. Expression pattern of genes as indicated after 48 h of BrdU treatment (100 μM) by microarray analysis. Data extracted from the microarray experiment is reported in Supplementary Fig. S1a (*n* = 2). The numbers indicate fold change in gene expression during BrdU treatment wrt Control (normalized as 1). **b** *CXCR4* gene expression analysis after DNA damage. HeLa and A549 cells were treated for 48 h with 100 μM BrdU followed by qRT-PCR analysis of *CXCR4* expression. The values were normalized to β-actin expression and then wrt control cells to calculate fold changes. Results shown are mean ± s.e.m. *p ≤ 0.05; ***p ≤ 0.001 (Student’s *t*-test; *n* > 3). **c** Gene expression analysis during IR-mediated DNA damage. Expression analysis of *CXCR4, p21*, and *IL8* after irradiation of HF-hTERT cells using qRT-PCR. The values were normalized to β-actin expression and then wrt control cells to calculate fold changes. Results shown are mean ± s.e.m. *p ≤ 0.05; **p ≤ 0.01 (Student’s *t*-test; *n* > 3). **d** Gene expression analysis during replicative exhaustion mediated DNA damage and senescence. Expression analysis of CXCR4 (left) and DDR genes *P21* and *P53* (right) in early (20 PDL) and late (40 PDL) passage MRC5 cells by qRT-PCR. The values were normalized to *β-actin* expression and then wrt control cells to calculate fold changes (*n* = 2). **e** Immunofluorescence and **f** surface expression analysis of CXCR4 receptor by flow cytometry. HeLa cells were probed for CXCR4 expression as per the protocol described in Materials and methods section. (magnification 40×; scale bar = 40 µm; *n* = 5 for **e** and *n* = 3 for **f**)

Considering evidence from literature where expression of CXCR4 has been seen in aggressive cancers^20^ and in cells which are senescent^16^ i.e., show persistent DNA damage response,^21^ we first performed an unbiased microarray analysis of HeLa cells after 48 h of BrdU (100 μM) treatment. We used BrdU as a chemotherapeutic agent for our experiments as it causes direct DNA damage by incorporating in the DNA in place of thymidine and also causes senescence when used at sub-lethal dose, which has been optimized previously.^16^ As anticipated, expression changes for genes classically associated with DDR and senescence such as *CDKN1A (P21)*, *IL6*, and *GNG11* was recorded (Fig. 1a and Supplementary Fig. S1a). The array data as well as validation in A549 and HeLa cells treated with BrdU for 48 h by quantitative PCR confirmed the increase in expression of *CXCR4* receptor (Fig. 1b) and decrease in proliferation as expected (Supplementary Fig S1b). The treated cells also stained positive for SA β-gal, a well-established marker for cellular senescence^6^ (Supplementary Fig S1c). Similar changes in *CXCR4* expression was confirmed in both HeLa and A549 cells, after treatment with another DNA damaging agent, doxorubicin for 48 h (Supplementary Fig S1d). To validate the applicability of our observations on non-cancerous primary cells, we also used HF-hTERT, an immortalized non-cancerous cell line,^22^ irradiated them with 14 Gy and harvested after 72 h to evaluate *CXCR4* levels. Irradiation enhanced the expression of *CXCR4*, which was concomitant to induction of *CDKN1A (P21)*, another DDR associated gene and *CXCL8* (*IL8)*, an inflammation associated gene (Fig. 1c). This confirmed that *CXCR4* upregulation during DNA damage is independent of cell type and DNA damage driver. As a control, no changes in levels of another GPCR, *CXCR7*, known to interact with *CXCR4* receptor^23^ was recorded (Supplementary Fig. S1e).

Furthermore, given that DNA damage and presence of DDR is also integral in replication exhaustion mediated cellular senescence,^24^ expression levels of *CXCR4* was also found to be enhanced in late passage primary fibroblasts, MRC5 (Fig. 1d, left panel), concomitant to other DDR markers such as *P21* and *TP53 (P53)*^6^ (Fig. 1d, right panel), reinforcing the positive correlation between DDR and CXCR4 induction. As anticipated the late passage cells were morphologically larger and senescent-like (Supplementary Fig. S1f). Immunofluorescence analysis and surface staining for CXCR4 confirmed that the observed transcriptional enhancement translates into higher protein levels in the damaged HeLa cells (Fig. 1e,f). We confirmed the specificity of the antibody recognizing CXCR4 by probing it on HeLa cells expressing CXCR4 siRNA (described previously) and here, loss of surface staining was recorded by flow cytometry (Supplementary Fig S1g).

### CXCR4 upregulation is mediated by the ATM kinase through HIF1α

**Fig. 2:**
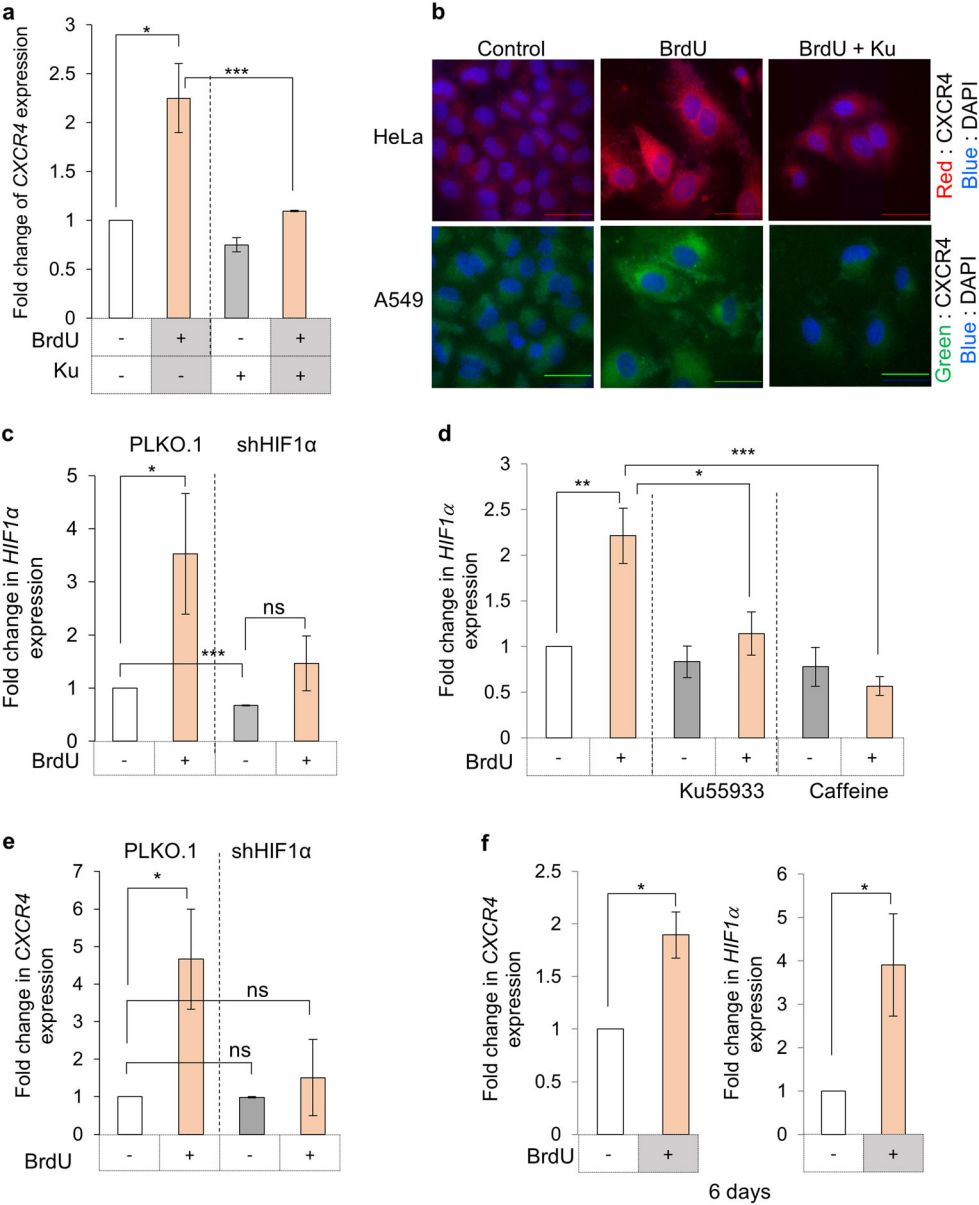
DNA damage response and CXCR4 expression. **a** Effect of ATM kinase inhibition on *CXCR4* levels during DNA damage. *CXCR4* expression analysis was performed by qRT-PCR in HeLa cells treated with BrdU and ATM kinase inhibitor as indicated. **b** Immunofluorescence analysis of CXCR4 expression. HeLa and A549 cells treated as indicated and probed for CXCR4 expression as described in Fig. 1E. Scale bar = 40 µm (*n* = 5). HeLa cells were probed with TRITC conjugated anti-CXCR4 antibody (red), while A549 cells with FITC–conjugated anti-CXCR4 antibody (green). **c** Analysis of *HIF1α* expression during DDR. Expression analysis of *HIF1α* in HeLa cells containing vector alone for HIF1α targeting shRNA after treatment with BrdU was performed by qRT-PCR (*n* = 3). **d** Effect of ATM kinase inhibition on HIF1α expression. HF-hTERT cells were treated with BrdU and treated with either Ku or caffeine. HIF1α expression was evaluated by qRT-PCR. (*n* = 3). **e** Analysis of *CXCR4* expression wrt *HIF1α* levels. *CXCR4* expression during BrdU induced DDR was analysed in HeLa cells stably expressing shRNA against *HIF1α* by RT-PCR (*n* = 3). **f** *CXCR4* and *HIF1*α expression analysis after prolonged BrdU treatment. Expression analysis of *CXCR4* (left) and *HIF1*α (right) was performed in HeLa cells treated with BrdU for 6 days by qRT-PCR and compared with untreated cells (*n* = 3). For all experiments results shown are mean ± s.e.m. *p* value ns < 0.5; *p ≤ 0.05; ***p ≤ 0.001 (Student’s *t*-test; *n* = 3)

Given that ATM kinase is primary regulator for DDR,^5,25^ we tested its role in CXCR4 induction by using a specific inhibitor, Ku-55933 (Ku)^26^ in presence of DNA damage (BrdU), and no enhancement in CXCR4 expression was observed at RNA level (Fig. 2a) and protein levels in HeLa and A549 cells (Fig. 2b). The effect of Ku-55933 on ATM kinase activity inhibition was confirmed by monitoring γH2AX levels, which were significantly lower in Ku treated cells (Supplementary Fig. S2a). This was also confirmed using caffeine (Caff), another inhibitor for ATM kinase and there also absence of CXCR4 induction at protein levels by flow cytometry analysis was recorded (Supplementary Fig. S2b).

We analyzed the changes in the expression levels of various transcription factors that are known to modulate CXCR4 expression and the level of HIF1α was induced in our microarray analysis (Supplementary Fig. S1a) post BrdU treatment to 1.4-fold compared to control cells. Hence, to identify the transcription factor downstream of ATM kinase, expression level of *HIF1α*,^27^ known to be a positive regulator of CXCR4 expression,^28^ were tested and found to be enhanced in damaged cells (BrdU treated) at both RNA (Fig. 2c) and protein levels (Supplementary Fig. S2c). In order to confirm that HIF1α induction is a result of DNA damage response and downstream ATM kinase activation post DNA damage, HeLa cells were treated with BrdU along with ATM kinase inhibitors, Ku or caffeine and analyzed for HIF1α expression after 48 h. During ATM kinase suppression, DNA damage failed to induce HIF1α expression (Fig. 2d and Supplementary Fig. S2d). To further evaluate its involvement, *CXCR4* expression was monitored in cells where HIF1α expression was suppressed using specific targeting shRNA (Fig. 2c and Supplementary Fig. S2c) and significantly lower induction of *CXCR4* was observed at both RNA (Fig. 2e) and protein levels (Supplementary Fig. S2e). These experiments established that during DDR, ATM kinase activation increases HIF1α levels, which in turn enhances *CXCR4* expression. CXCR4 expression remained elevated for prolonged period after BrdU treatment (Fig. 2f, left panel), suggesting that *CXCR4* is part of the gene set which is upregulated during DNA damage response, similar to *P21, P53, CDKN2A (P16)*, etc.^6^ Concomitant to this we also recorded elevated levels of HIF1α in cells, which are in senescent state for a long time (Fig. 2f, right panel).

### Activation of CXCR4 signaling in presence of DDR suppresses its pro-proliferative role

**Fig. 3:**
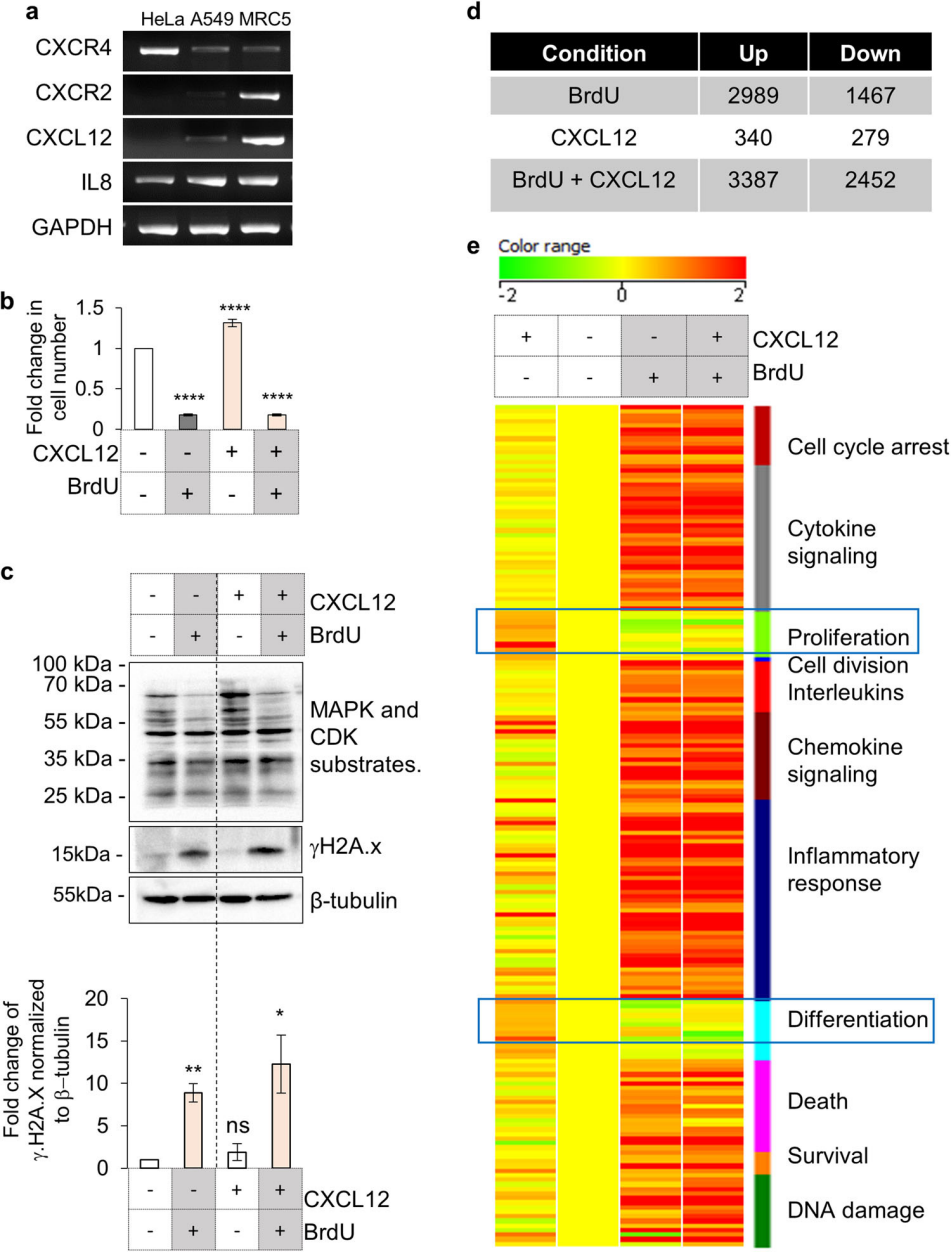
Analysis of effects of CXCR4 activation in damaged cells. **a** Gene expression analysis to evaluate transcription of *CXCR4* and *CXCR2* and respective ligands by PCR. Expression analysis of mentioned genes was performed in HeLa, A549 and MRC5 cells (Table S2). **b** Effect of CXCL12-CXCR4 axis on cellular proliferation. HeLa cells, with or without CXCL12 (200 ng/ml) or BrdU were counted after 72 h of treatment. The *Y*-axis represents fold change of proliferative index wrt undamaged unstimulated cells. Results are mean ± s.e.m. ****p ≤ 0.0001 (Student’s *t*-test; *n* > 10). **c** Analysis for γH2A.x and MAPK/CDK substrate phosphorylation on activation of CXCR4 receptor. HeLa were treated with CXCL12 (200 ng/ml) in presence or absence of 100 µM BrdU for 48 h and the levels of MAPK-CDK substrates (top), phospho-H2Ax (middle panel) and β-tubulin (bottom) were analyzed by western blotting. Quantitation of γH2Ax levels are shown in the graph (*n* = 3). **d** Microarray analysis of gene expression changes in response to CXCL12 treatment. Table shows number of gene up or downregulated in comparison to untreated HeLa cells in presence of BrdU, CXCL12 or BrdU with CXCL12 after 48 h (*n* = 2). **e** Heat map of functional clusters in microarray data. Genes differentially regulated in presence of BrdU or CXCL12 alone or with BrdU along with CXCL12 (as indicated) were clustered into indicated families and heat map was generated based on fold changes (*n* = 2)

Given that CXCR4 is a GPCR and its activation is necessary for identifying its role, we evaluated the expression of *CXCR4* and its ligand *CXCL12* in various cell lines, viz. HeLa, A549 and MRC5 (a primary fibroblast line). At the same time we also probed for expression levels of another GPCR, *CXCR2*, previously reported to be a regulator of DDR dependent cellular senescence^14^ along with its ligand, *IL8*. Interestingly, by gene expression analysis we recorded *CXCR4* and *IL8* expression in all cells, but no expression of *CXCR2* or *CXCL12* was observed in HeLa cells (Fig. 3a).

It has been proposed that deleterious impact of CXCR4 expression in cancer cells is due to its pro-proliferative role. Also it is known that during development or in non-damaged cells, CXCL12-CXCR4 axis triggers both proliferation and differentiation.^29^ Given that HeLa cells do not express CXCR4 ligand, CXCL12, they provided a unique system for assessing the effect of CXCR4 activation without any interference from endogenous ligand.^30^ When HeLa cells were stimulated with CXCL12, in presence of DNA damaging agents, CXCR4 activation dependent proliferation and associated MAPK/CDK substrate phosphorylation was found to be absent (Fig. 3b and c, top panel). Furthermore, activation of CXCR4 in presence of BrdU further increased γH2A.x levels (Fig. 3c, middle panel), indicating enhanced DDR. Comparative gene expression analysis with or without CXCL12, revealed that the presence of CXCL12 with BrdU suppressed the cellular proliferation responses and enhanced the DDR, cell cycle arrest, cytokine/chemokine signaling, inflammatory response and pro-survival pathways after 48 h of treatment (Figs. 3d, 3e and Supplementary Fig. S3a). All the responses after stimulation were higher than the cells treated with BrdU alone and the findings also suggested that DDR pathways take precedence over proliferative cues. Gene expression changes unique to CXCL12 and BrdU treated cells were further analyzed using DAVID^31^ and here also some genes involved in biological processes such as immune response and migration were found to be upregulated whereas those involved in processes like cell cycle were downregulated (Supplementary Fig. S3b). Validation of upregulated DDR and inflammation genes like *IL8, IFI27, DGKA, WISP2, TAC3*, and *P21* by qRT-PCR (Supplementary Fig. S3c) confirmed that the activation of CXCR4 during DDR strengthens injury and inflammatory responses, compared to cells which are exposed only to BrdU without CXCL12.

### CXCR4 activation induces DDR dependent inflammation by reducing cAMP levels

**Fig. 4:**
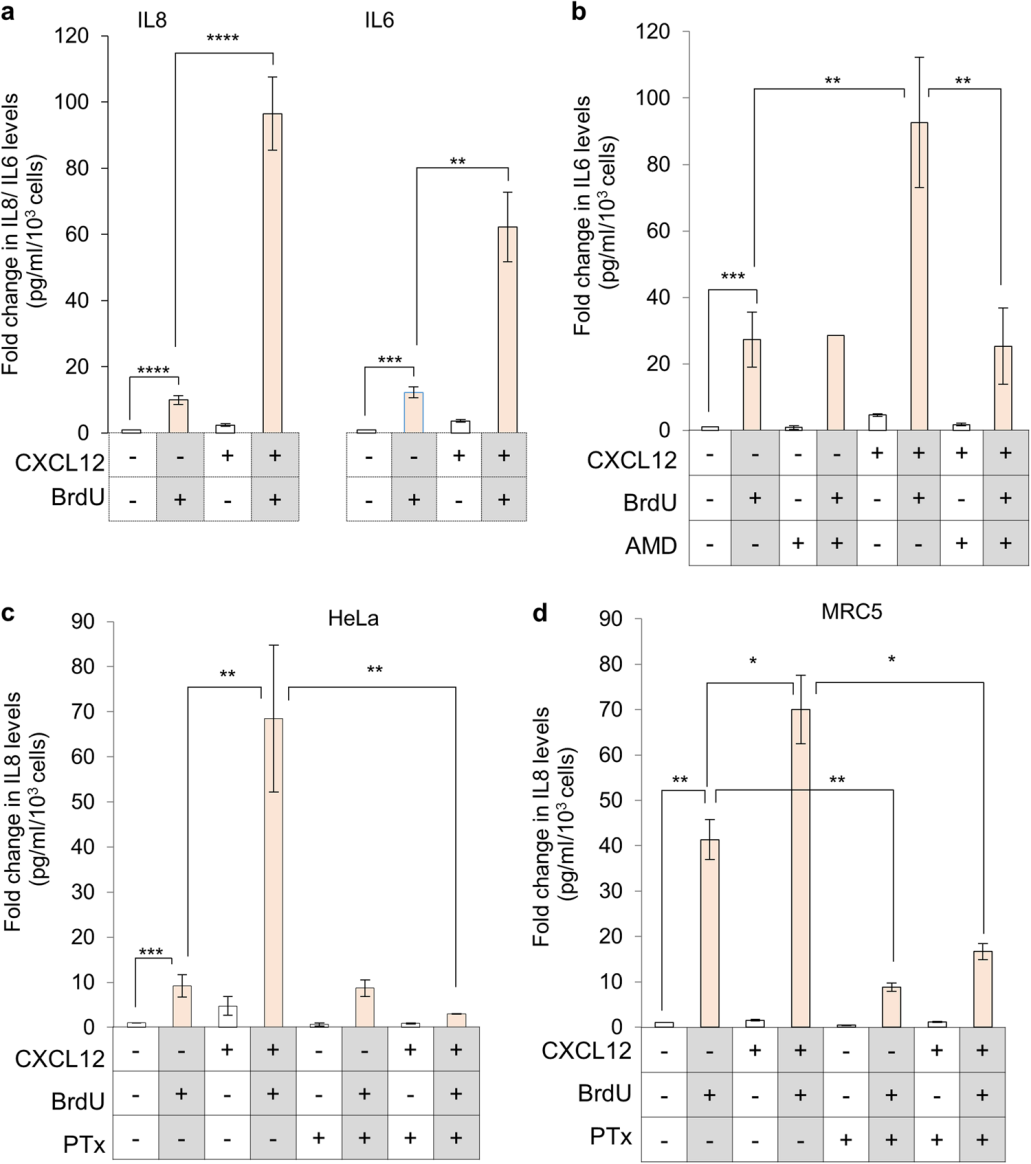
CXCR4 receptor regulates DNA-damage associated inflammation. For all experiments, media from treated cells (as indicated), was used for sandwich ELISA for determining levels of IL8 or IL6 pg/ml secreted per 10^3^ cells, represented as fold change over control untreated cells. **a** Effect of activation of CXCR4-CXCL12 signaling during DNA damage on IL8 and IL6 cytokine secretion (*n* > 6). **b** Effect of CXCR4 inhibition (AMD treatment) on IL6 production from senescent cells post-CXCL12 stimulation. Cells were treated with various compounds as indicated and IL6 levels in supernatant were analysed (*n* = 3). **c** Effect of inhibition of Gαi by PTx treatment. Comparison of IL8 levels between HeLa cells treated with BrdU; BrdU + CXCL12 or BrdU + CXCL12 in presence of pertussis toxin (PTx) (*n* = 3). **d** Effect of inhibition of Gαi by PTx treatment on MRC5 cells. Comparison of IL8 levels in MRC5, primary cells treated with BrdU; BrdU + CXCL12 and BrdU + CXCL12 in presence of PTx (*n* = 3). For all experiments, results are represented as mean ± s.e.m. *p ≤ 0.05, **p ≤ 0.01, ***p ≤ 0.001, ****p ≤ 0.001 (Student’s *t*-test)

One of the hallmarks of DDR is enhanced inflammation, observed during anti-cancer therapies^32,33^ as well as senescence,^6,34^ which is known to be negatively regulated by cAMP.^35^ Given that CXCR4 receptor activates Gαi/o subunit, which on activation inhibits adenylyl cyclase activity and reduces cAMP levels, it is anticipated that its activation will increase inflammation. In line with this hypothesis, the levels of senescence associated inflammatory molecules IL6 and IL8 were found to be higher in HeLa cells treated with BrdU in presence of CXCL12 compared to cells where CXCR4 receptor was not activate (Fig. 4a). A similar effect was recorded in A549 cells (Supplementary Fig. S4a) and this enhancement was abrogated in the presence of CXCR4 antagonist, AMD3100 (Fig. 4b). Inhibitory activity of AMD3100 was confirmed by monitoring Ca^+2^ release on CXCL12 stimulation, which was absent in AMD treated cells (Supplementary Fig S4b). It was interesting to note that the CXCR4 dependent inflammatory response was absent during early time point (24 h after BrdU treatment) when the cells are not yet senescent (Supplementary Fig. S4c). This was a critical observation, demonstrating that while CXCR4 induction and activation enhances inflammation in the damaged cell, it is initiated only after the DNA damage has been detected and cellular senescence as the fate is decided, a process which is independent of CXCR4. Treatment with Pertussis toxin (PTx), which inactivates Gαi/o subunits,^36^ suppressed increase in IL8 levels in HeLa (Fig. 4c) as well as in primary MRC5 cells (Fig. 4d) indicating a G-protein dependent role of CXCR4 receptor in the inflammatory response.

**Fig. 5:**
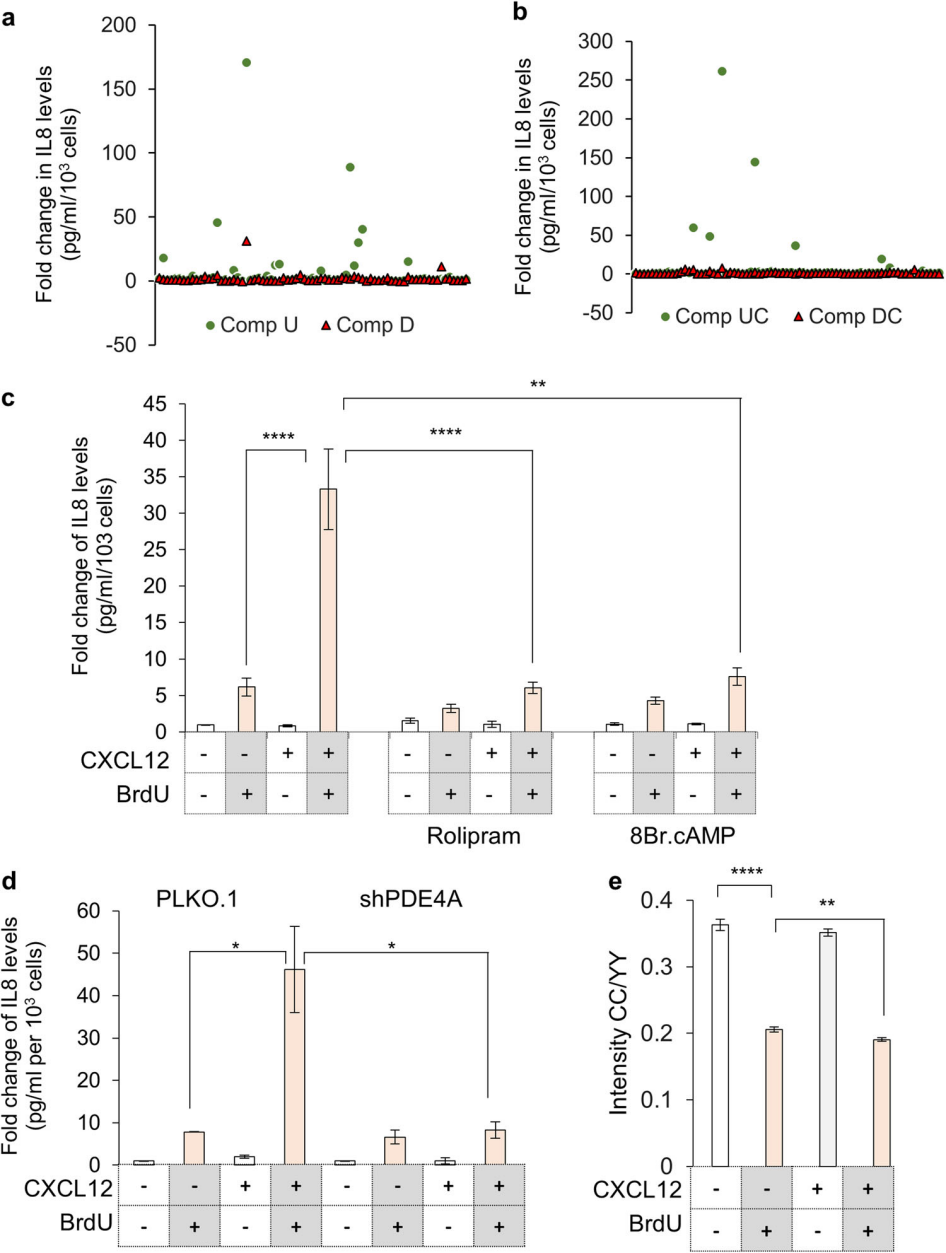
Identification of signaling cascade involved in CXCR4-activation dependent enhanced inflammation during DNA damage. **a** Screening for compounds modulating DNA damage associated inflammation. Scatter plot for individual compounds in LOPAC plate 1 representing fold change of IL8 secretion (through ELISA) when compared to untreated (Comp U, circle) or damaged cells (Comp D, triangle). **b** Scatter plot of compounds modulating CXCR4 activation dependent enhanced DNA damage-associated inflammation. Scatter plot for individual compounds from LOPAC plate 1, representing fold change of IL8 levels (through ELISA) compared to CXCL12 stimulated cells which are untreated (Comp UC, circle) or in presence of DNA damage (Comp DC, triangle) is shown. **c** Role of cAMP modulators on CXCR4 dependent enhanced inflammatory response. The media from treated HeLa cells (as indicated) was used for determining IL8 levels compared to untreated control cells (*n* = 3). **d** Effect of PDE4A expression knockdown on CXCR4 activation dependent inflammation. The media from treated and untreated HeLa cells transfected with shRNAs as indicated was used to determine levels of IL8 (*n* = 4). **e** Measurement of cAMP levels in CXCL12 treated damaged cells. FRET ratios recorded by measuring CC/YY intensity changes in ICUE3 reporter in HeLa cells treated as indicated (Cell images are shown in Supplementary Figure S5d). For all experiments results are shown as mean ± s.e.m. *p ≤ 0.05; **p ≤ 0.01; ***p ≤ 0.01; ****p ≤ 0.001 (Student’s *t*-test; *n* = 3)

To further characterize the signaling cascade underlying the CXCR4-mediated and DDR dependent enhanced inflammation, a pharmacologically active compound library (LOPAC, Sigma) was screened, to identify molecules which specifically suppress CXCR4-dependent inflammation enhancement. It is essential to point out here that the CXCR4 activation only enhances the inflammation over and above the basal inflammation during DDR, which is observed in all damaged cells. In the LOPAC screen, cell proliferation and inflammation levels changes were evaluated in presence of compound alone; compound along with BrdU (Fig. 5a) and in presence of CXCL12 with or without DNA damage (BrdU) (Fig. 5b). While some compounds, such as Budesonide, Reserpine and p38 MAPK inhibitors completely blocked inflammation, a few significantly affected the CXCL12-dependent inflammation enhancement without much affecting the basal DDR-dependent inflammation. Inhibition of cAMP specific phosphodiesterase 4A (PDE4A) using Rolipram, also significantly suppressed the CXCR4-dependent inflammation in a similar way as non-hydrolysable cAMP analog, 8-bromo cAMP (Fig. 5c, Supplementary Fig. S5a and S5b). The role for PDE4A was further confirmed using shRNA specifically suppressing *PDE4A* expression (Supplementary Fig. S5c) and here also inflammation enhancement after CXCR4 stimulation was reduced (Fig. 5d). To validate that the levels of cAMP affects the inflammation, a FRET based cAMP sensor, ICUE3 was utilized,^37^ where FRET ratio is inversely proportional to cAMP levels. As anticipated, we recorded high FRET signal in damaged cells indicative of low cAMP, in comparison undamaged cells (Fig. 5e and Supplementary Fig. S5d). Presently no direct regulation of PDE4 through CXCR4 is known and we also did not record any change in PDE4A gene expression in any of the treatment conditions (Supplementary Fig. S5e). In sync with this, our experiments also suggest that if there is a reduction in the levels of cAMP, which probably occurs through concerted action on both Gαi/o protein activation mediated adenyl cyclase inhibition and activity of PDE4 protein, enhanced inflammatory response is recorded. This is lost when either of the responses are blocked, by either CXCR4 antagonist (which prevents Gαo/i activation) or PDE4A inhibitor, leading to an enhancement in cAMP levels. Increase in cAMP levels above a threshold suppress enhanced inflammation, hence similar effects from PDE4 inhibitor or CXCR4 inhibitor were recorded.

### CXCR4 upregulation is essential for DDR associated inflammation

**Fig. 6:**
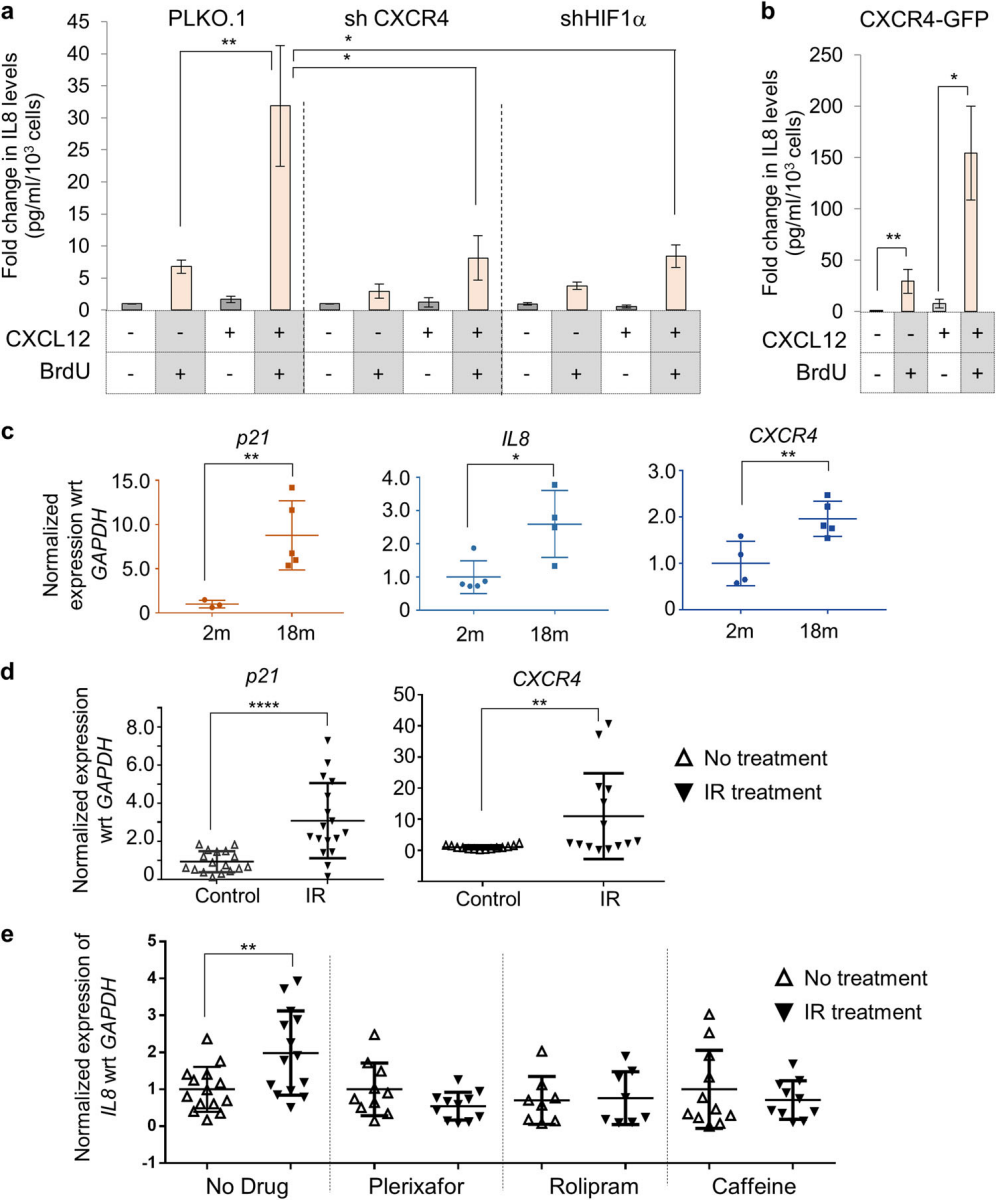
Role of CXCR4 receptor and its upregulation in DNA-damage associated inflammation. **a** Effect of CXCR4 receptor level changes on enhanced inflammation during DNA damage. IL8 levels measured from HeLa cells where expression of CXCR4 (left) or HIF1α (right) was knocked down using gene specific shRNAs. Experiments were performed as described in Fig. 5a. **b** Analysis of inflammatory response in CXCR4-OE cells. CXCR4-GFP cells were treated with BrdU or BrdU + CXCL12 and IL8 ELISA was performed. **c** Analysis of gene expression changes during organismal aging. Quantitative RT-PCR analysis for the expression of *p21*, *IL8* and *CXCR4* in liver tissues of 2-months and 18-months old BALB/c mice. The values were normalized to GAPDH expression levels. **d** Analysis of gene expression changes during IR mediated damage in C57BL/6 mice. Quantitative RT-PCR analysis for the expression of *p21* and *CXCR4* in irradiated animals. **e** Analysis of IL8 levels during irradiation and in response to various inhibitors. Animals were treated with Caffeine (Caff), Plerixafor (Plx) or Rolipram (Roli) alone or with these compounds and irradiation. Gene expression changes for IL8 was tested in liver as mentioned in Fig. 6c. For all experiment, *n* > 5 and as indicated in the figure panels. *p*-value or significance was determined by ANOVA and Bonferroni’s multiple comparison test. Significance (*p* value) is represented as *, *≤0.05, **≤0.01, ***≤0.001, and ****≤0.001 and ns, where >0.05 for “not significant”

HeLa cells stably expressing *CXCR4* targeting shRNA did not show an enhanced inflammation when treated with CXCL12 in presence of BrdU, confirming that CXCR4 is essential for inflammation enhancement (Fig. 6a, left panel and Supplementary Fig. S6a, left panel). Next, the requirement of CXCR4 upregulation was evaluated by suppressing expression of upstream transcription factor, HIF1α and here too inflammation enhancement was abrogated (Fig. 6a, right panel and Supplementary Fig. S6a, right panel), indicating that upregulation of CXCR4 receptor is essential. In a parallel experiment, HeLa cells overexpressing the CXCR4 receptor showed additional increase in the inflammatory response during DNA damage (~200 fold) (Fig. 6b), which in toto indicated that CXCR4 expression level drives inflammation enhancement in presence of DDR. Validation of GFP-tagged CXCR4 receptor activity was done by monitoring receptor internalization post CXCL12 stimulation (Supplementary Fig S6b). Overall, the transition of cell into senescent state which is associated with CXCR4 upregulation, is critical for the enhancement in the inflammatory axis.

### Suppression of CXCR4 expression induction or activation in vivo abrogates inflammation post DNA-damage

Tissue injury due to DNA damage is one of the primary cause of physiological malice during anti-cancer therapies^38^ as well as during chronological aging, where an increase in DNA damage with time has been recorded.^4,39^ In this context, first we compared gene expression changes between 2-month and 18-month-old BALB/c mice to probe if changes in DNA damage associated genes are recorded. We observed induction in levels of *p21* gene, a classical marker associated with aging; *Cxcl15 (IL8)*, an inflammatory cytokine as well of *Cxcr4* receptor in older mice (Fig. 6c), suggesting that CXCR4 can serve as a marker for DNA damage at both cellular and organismal level. Similar observations were made in 4–6 weeks old C57BL6 mice subjected to sub-lethal dose (6 Grays) of ionizing radiation (IR), which causes significant DNA damage^40,41^ and here also upregulation of *p21*, *Cxcr4* and *Cxcl15 (IL8)* was recorded (Fig. 6d), confirming that the response underlying DNA damage and aging is similar. To specifically confirm presence of DNA damage response, we probed for phospho-H2A.X levels through western blotting and recorded elevated levels in physiologically aged 18-month-old mice compared to 2-month-old mice as well as in IR-treated mice compared to untreated (Supplementary Fig. S6c, densitometric data is shown). Next, using the IR-mediated DNA damage mouse model, the effect of CXCR4-CXCL12 signaling on inflammation and associated tissue injury was analyzed. Interestingly, changes in levels of *Cxcl12* remained unaltered during natural ageing as well as IR-induced accelerated ageing (Supplementary Fig. S6d). For evaluating role of ATM kinase and CXCL12-CXCR4 axis in damage-induced inflammation, mice were irradiated while being treated with caffeine (Caff), which inhibits ATM kinase^25^ or with Plerixafor (Plx), a CXCR4 antagonist^42^ or with Rolipram (Roli), PDE4A inhibitor,^43^ based on the *in cellulo* findings reported above. Treatment with these inhibitors suppressed the DDR mediated upregulation of IL8 in the liver (Fig. 6e), indicative of reduction in inflammation, a hallmark of tissue injury through the same DDR cascade reported above. We also evaluated expression status of IL10, another cytokine which has been linked to senescence pathology^44^ which was also induced after IR mediated injury and the induction was lost in the presence of caffeine, Plerixafor or Rolipram (Supplementary Fig. S7a). Further, in sync with the cascade mapped in *in cellulo* studies (above), treatment with caffeine suppressed *Cxcr4* induction as well as *IL8* levels (Supplementary Fig. S7b).

When various tissues of these animals were examined histologically, it was observed that pretreatment with Rolipram or caffeine reduced tissue injury associated with DNA damage on irradiation. In liver, animals subjected to IR showed impaired architecture of sinusoids and canalicular architecture, presence of hemorrhage and portal hylinization, which was absent in animals treated with any of the inhibitors (Supplementary Fig. S7c). Histological examination of lung revealed presence of IR mediated injury, marked by persuasive alveolar thickening, presence of hemorrhage and fibroblastic foci (Supplementary Fig. S7c and Supplementary Table 3), which was significantly abrogated in presence of Rolipram. We also noted that the size of spleen, which was *per se* smaller in the IR treated animals, was not restored on treatment with either caffeine or Plerixafor (Supplementary Fig. S7d). Overall, using irradiated mouse model, we recorded suppression of inflammation, tissue injury and DNA damage in liver (where maximum impact of IR was recorded), when CXCR4 signaling, its upregulation or cAMP level increase was inhibited.

These physiological findings make CXCR4 upregulation during DDR highly relevant and demonstrate that the CXCR4-CXCL12 signaling cascade is a critical regulator of inflammation associated with DNA damage. The findings also allow us to propose this pathway as a potential target for co-therapy during anticancer treatment regimens, primarily to suppress inflammation associated complications including metastasis. These observations also demonstrate existence of a local “inflamma-modulatory” response, establishing an inflammatory cytokine gradient necessary for homing of immune cells only to the damaged cells (Supplementary Fig. S7e), which is perhaps more systemic in nature compared to DDR alone response, which could be more local.

## Discussion

GPCRs regulate diverse cellular processes, both in normal and pathological conditions and because of membrane association, they are also attractive therapeutic targets. Targeting Histamine receptors, which are GPCRs through anti-histamines (antagonists) has been very successful in suppressing topical inflammation.^45^ Similarly other receptors which sense endogenous inflammatory ligands such as leukotriene and prostaglandin have also been targeted for developing anti-inflammatory therapies.^45^ However, no clear role for any specific GPCR in directly regulating inflammation during DNA-damage or aging has been proposed, even though it is known to be regulated by many kinases such as p38, MEK, Jun etc.^34^ In the present study, we describe a previously unknown role for CXCR4 signaling in DNA damage associated inflammation. Enhancement in CXCR4 receptor expression has been recorded in highly aggressive cancers,^20^ post-chemotherapy,^46^ in cellular senescence as well as in physiologically aged neutrophils.^17^ We found that CXCR4 expression is enhanced during any stress-response which activates DDR, be it in chemotherapy, radiotherapy or in cells which undergo replicative senescence, thereby proposing a potentially universal role for this receptor in DDR.

Mechanistic analysis of CXCR4 upregulation revealed that it is a part of DNA damage response pathway and can serve as a marker of DNA damage encountered by a cell, similar to histone H2A.X phosphorylation or p21, p16 upregulation.^6^ Further, we believe that our findings can provide explanation for observed CXCR4 upregulation in many cancers^15^ especially after anticancer therapy^46^ where considerable DDR is generated. Pathway analysis confirmed that CXCR4 upregulation is a part of DDR, as it is mediated by activation of ATM kinase and the transcription factor, HIF1α, which was also found to be upregulated as a part of DDR. Interestingly, the presence, absence or induction of CXCR4 expression by itself was not essential for DNA damage response, indicative of its role in post-DDR signaling events only, similar to many inflammatory molecules whose levels increase during aging, but they themselves may not drive aging but positively reinforce it.^47,48^

Analysis of this upregulation revealed that the activation of CXCR4 receptor during DNA damage reduces cAMP, which drives inflammation through the typical PKA, p38, NFκB cascade^49^ and suppresses pro-proliferative changes typically associated with CXCR4 receptor.^50^ Given that CXCR4 is ubiquitously expressed and CXCL12 is a homeostatic chemokine,^51^ our findings demonstrate that the inflammatory role of this cascade is initiated only upon CXCR4 induction during DDR, which increases sensitivity of the damaged cells to its ligand CXCL12. In a nutshell, we thus report existence of a mechanism of gradient sensing, wherein damaged cells increase the expression of the CXCR4 receptor, thereby facilitating enhancement of signaling response (super sensitivity) to the ligand, CXCL12 and potentiate inflammation through further suppression of cAMP levels. Similar enhanced sensitivity and downstream effector activation through increase in receptor expression has been observed for oxytocin receptor in myometrium, where enhanced signaling facilitates changes needed in uterus for initiating labor.^52^ Similarly, on chronic antagonist treatment for μ-opioid receptors, receptor upregulation has been observed, which makes cells super-sensitive to agonist.^53^

Overall, a GPCR-mediated signaling pathway for regulating DNA-damage response-dependent inflammation was identified, wherein a communication between damaged cells and stromal factors generate a local inflammatory response to facilitate recruitment of immune cells for clearance. In case of damage during aging or anticancer therapy, where the damage is systemic in nature and associated inflammation possibly leads to metastasis of cancer cells, inhibition of this axis should facilitate improvement in quality of life and better chemo or radio-therapeutic outcomes.

## Methods

### Cell culture, treatment, shRNA knockdown and induction of DNA damage

HeLa, A549, MRC5 cells (ATCC, USA) and HF-hTERT (gift from Dr. A. Rangarajan, IISc.) were grown in DMEM with 10% FBS at 37 ^o^C; 5% CO_2_ and were treated with various agents as mentioned. For ATM kinase, CXCR4, HIF1α and PDE4A knockdown, validated pooled shRNA from TRC library (Sigma Aldrich, USA; sequences in Table S1) were transfected to generate stable knockdown cells using puromycin (3 µg/ml). The knockdown efficiency was verified by qRT- PCR analysis using gene specific primers (sequences in Table S2) and by western blotting or surface staining.

To induce DNA damage, the cells were treated with 5-bromo deoxyuridine, (Sigma, USA) (100 µM) (2); or doxorubicin (Sigma, USA) (0.1 µM) or 14 Gy of ionizing radiation (Blood Irradiator B1 2000) for indicated time durations. The treated and untreated cells were processed for various experimental analyses as described. Ku55933 (EMD Biosciences, USA) was used at 10 µM concentration for durations as indicated. Purified endotoxin-free CXCL12 recombinant human protein (Thermo Fisher Scientific, USA; Cat No.10118HNAE25) was used at 200 ng/ml; CXCR4 antagonist, AMD3100 (Sigma Aldrich, USA; 1 mg/ml in water) at 1 µg/ml; PTx (Merck Inc., USA), Rolipram (Cayman Chemical Co., USA; Cat No. 10011132), Plerixafor (Cayman Chemical Co., USA; Cat No. 1001132) and 8-Bromo-Cyclic AMP (sodium salt) (Sigma Aldrich, USA; B7880) and all the molecules in the LOPAC library (Sigma Aldrich, USA) were used at a concentration of 10 µM.

### Gene expression profiling

Total cellular RNA from cell lines was isolated using TRI reagent (Sigma, USA) and cDNA synthesis was performed using cDNA Reverse Transcription Kit followed by quantitative expression analysis using SYBR Green qPCR Kit (Thermo Fisher Scientific, USA) as per manufacturer’s instructions. Expression levels of β-actin and GAPDH were used to normalize the expression levels. RotoGene-Q real-time instrument and associated software was used for data and melting curves analysis. Primers used are mentioned in Table S2. Microarray analysis was done using established protocols and details are provided as supplementary data. GEO accession number for the data is GSE93568.

### Western blot and ELISA analysis

For western blotting, 50–100 µg of total protein lysate from cells was used. Detailed protocol and antibodies used are described in supplementary data. For estimating extracellular levels of various cytokines using media collected from treated cells as indicated, BD OptiEIA™ Human IL8, IL6 and TGFβ ELISA kits (BD Biosciences, USA) were used as per manufacturer’s instructions. The cells were counted to normalize the amounts to 10^3^ cells and it was ensured that the raw values obtained are within the dynamic range of the assay. All the blots were processed in parallel and were derived from the same experiment. Densitometric analysis of the blots was performed using ImageJ and all the changes in the expression as normalized wrt the expression levels of β-actin or as indicated.

### Immunofluorescence analysis for CXCR4 expression

For analysis of expression levels by microscopy, cells were fixed in 4% PFA, probed with anti-CXCR4 antibody (1:100) (Sigma C8352 or ThermoFisher Scientific, Cat No. MHCXCR404) for 1 h, followed by TRITC conjugated IgG antibody (anti-rabbit, 1:400). The cells were counterstained with DAPI and imaged. For surface expression analysis using flow cytometry, treated cells were detached using EDTA and live cells were incubated with 10 µg/ml of anti-CXCR4 antibody (Sigma, C8352) on ice for one hour, followed by Alexa Fluor 488-tagged secondary antibody (CST, 4412S; 1:500) for 30 min at 4 °C. The cells were then washed and analysed by flow cytometry using 488-nm laser for detecting surface expression levels. For all experiments, FITC-conjugated isotype control antibody was used to set the background. As a control for antibody, cells where CXCR4 expression was suppressed using siRNA^54^ was also used.

### cAMP measurements

HeLa cells were transfected with FRET-based cAMP reporter ICUE3 (gift from Jin Zhang; Addgene plasmid #61622)^37^ and were treated with various agents as mentioned and imaged after fixing with 4% PFA. FRET was quantitated using an Olympus IX83 inverted fluorescence microscope. Images were acquired in CFP, YFP and CFP-YFP FRET channels at same exposure and gain settings and analyzed using ImageJ software.

### Animal experiments

All experiments were performed as per guidelines from Control and Supervision of Experiments on Animals (CPCSEA), Government of India and with approval from Institutional Animal Ethics Committee (IAEC), IISc, Bangalore. For organismal ageing, 2 and 18-months-old female BALB/C mice obtained from National Institute of Nutrition (Hyderabad, India) were used. These mice were perfused with PBS before harvesting tissues for analysis. The mice used for irradiation experiments, weighed approximately 20–25 g and to induce DNA damage, 4–6 weeks old female C57BL/6 mice were irradiated with 6 Gy units of γ-radiation (Blood irradiator BI2000). Intraperitoneal injection of CXCR4 antagonist plerixafor (Cayman Chemical, USA), ATM kinase inhibitor, caffeine and Rolipram was given at a final concentration of 7.5, 50, and 0.5 mg/kg body weight respectively. After 3 days of irradiation, the animals were sacrificed and various tissues (as indicated) were collected for RNA isolation and histological analysis. For histology, tissue sections were fixed in 4% PFA and processed in a pathological laboratory as per established protocols and stained with hematoxylin and eosin. Blinded histological scoring was performed as per the established guidelines and protocols after analysis of samples at 10x objective using an Olympus CX21i upright microscope.

### Statistical analysis

For cell-based experiments, biological triplicates or more were used. And for animal experiments 5 or more animals were used per group. And all n’s are mentioned in the figure legends. Microsoft Excel was used for generating bar graphs and contour map. For all experiments, results are represented as mean ± s.e.m. For statistical analysis Students *t*-test (for most in vitro experiments in which two groups are compared), Mann–Whitney test (for animal experiments comparing two groups) and Bonferroni’s multiple comparison test (for analysis involving multiple groups) were used and a horizontal line on the graphs indicates comparisons across two sets of data sets. Significance (*p* value) is represented as *, where *≤0.05, **≤0.01, ***≤0.001, and ****≤0.001 and ns, where >0.05 for “not significant”. The *p* values were calculated with respect to the untreated cells or animals in all cases unless mentioned otherwise.

## Electronic supplementary material

SUPPLEMENTAL MATERIAL

## Data Availability

GEO accession number for the microarray data is GSE93568 and all other relevant data are available from the authors.

## Electronic supplementary material

**Supplementary information** accompanies the paper on the *npj Aging and Mechanisms of Disease* website (10.1038/s41514-018-0028-0).
