# Supplementary material for: Clampdown of inflammation in aging and anticancer therapies by limiting upregulation and activation of GPCR, CXCR4
Source: NPJ Aging Mech Dis. 2018 Aug 30;4:9. doi: 10.1038/s41514-018-0028-0 (PMC6117261; doi:10.1038/s41514-018-0028-0)

Supplementary Fig 1.

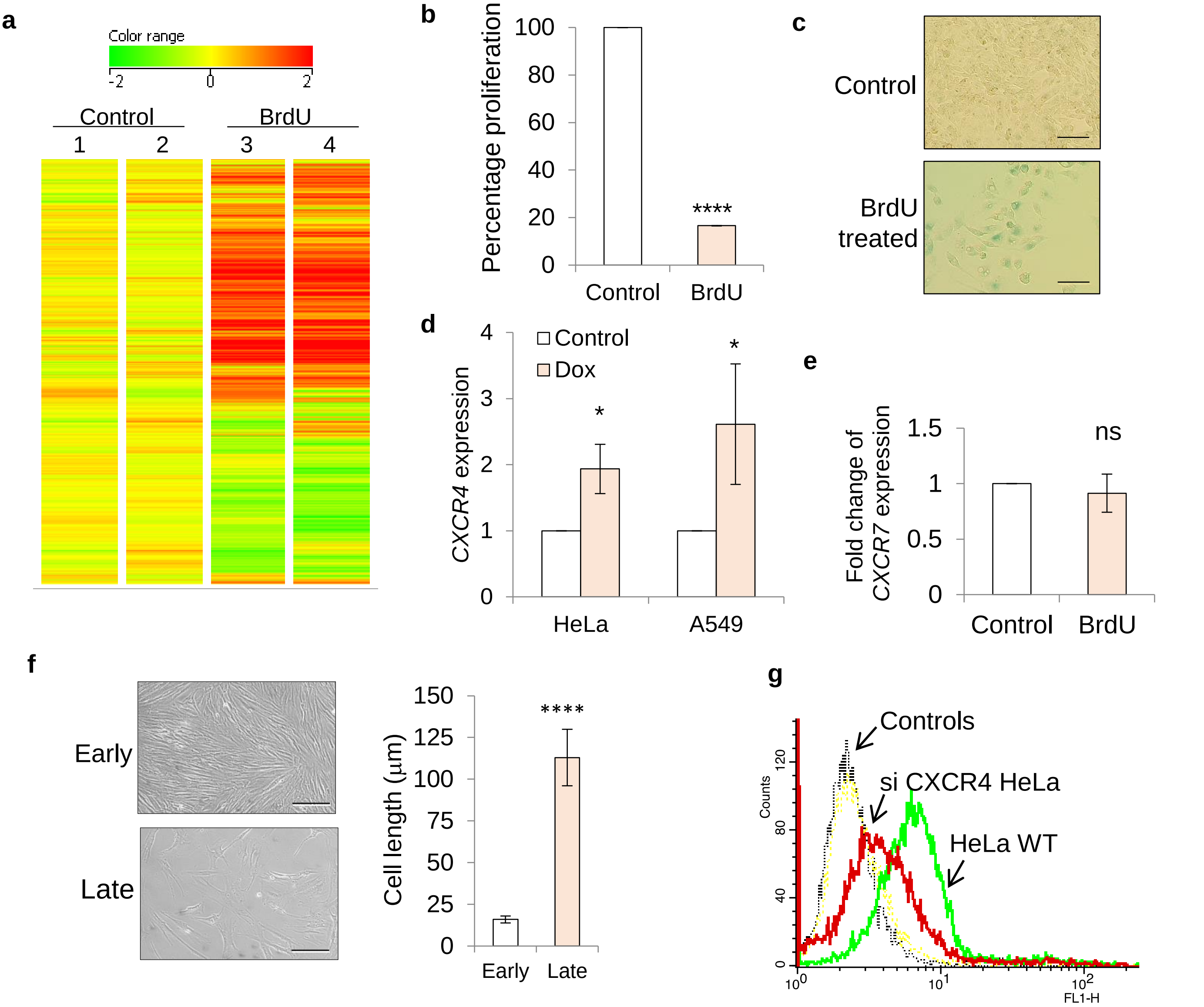

Fig. S1 Gene expression profiling and physiological characterization of cellular senescence. (a) Heat map showing differential genes expression between untreated cells and treated with DNA damaging agent, BrdU. Microarray based gene expression comparison was done between untreated HeLa cells (samples 1, 2) and those treated with BrdU for 48 hours (samples 3,4). Heat map generated based on expression changes is shown. The color range depicts fold change of genes expressed. (**b**) **Proliferation rate analysis for BrdU treated cells.** Equal number of HeLa cells were seeded overnight and treated with BrdU for 48 hours and cells numbers were counted after treatment. Data shown represents percentage proliferation wrt control cells (100). **(c) SA β-gal staining of BrdU treated HeLa cells.** Magnification, 20x, scale bar = 100µm. Left panel, control cells and right, BrdU treated cells (n=3). **(d) *CXCR4* expression analysis in senescent cells generated by doxorubicin treatment**. qRT-PCR analysis for *CXCR4* levels in HeLa and A549 cells treated with doxorubicin, normalized to *β-actin* (n=3). (**e**) **Expression of *CXCR7* receptor.** RT-PCR to analyze expression changes in *CXCR7*, normalized to *GAPDH* expression and then wrt to the control cells to calculate fold changes is shown (n=3). (**f**) **Morphological changes during replicative exhaustion.** MRC5 primary fibroblast cells were cultured for multiple passages and morphological features were evaluated for early and late passage cells. Magnification 10x, scale bar = 100 µm. Top panel, early and bottom panel, late passage cells. Right, quantification for cell length (measured across nucleus through shortest distance). **(g) Analysis CXCR4 expression by flow cytometry.** HeLa cells were transfected with plasmid containing siRNA sgainst CXCR4 and surface staining for CXCR4 was analyzed using flow cytometry to compare with untransfected HeLa cells expressing CXCR4. Results are shown as mean ± s.e.m. * p value ≤ 0.05; **** p value ≤ 0.0001 (Student's t-test; n=3).

Supplementary Fig 2.

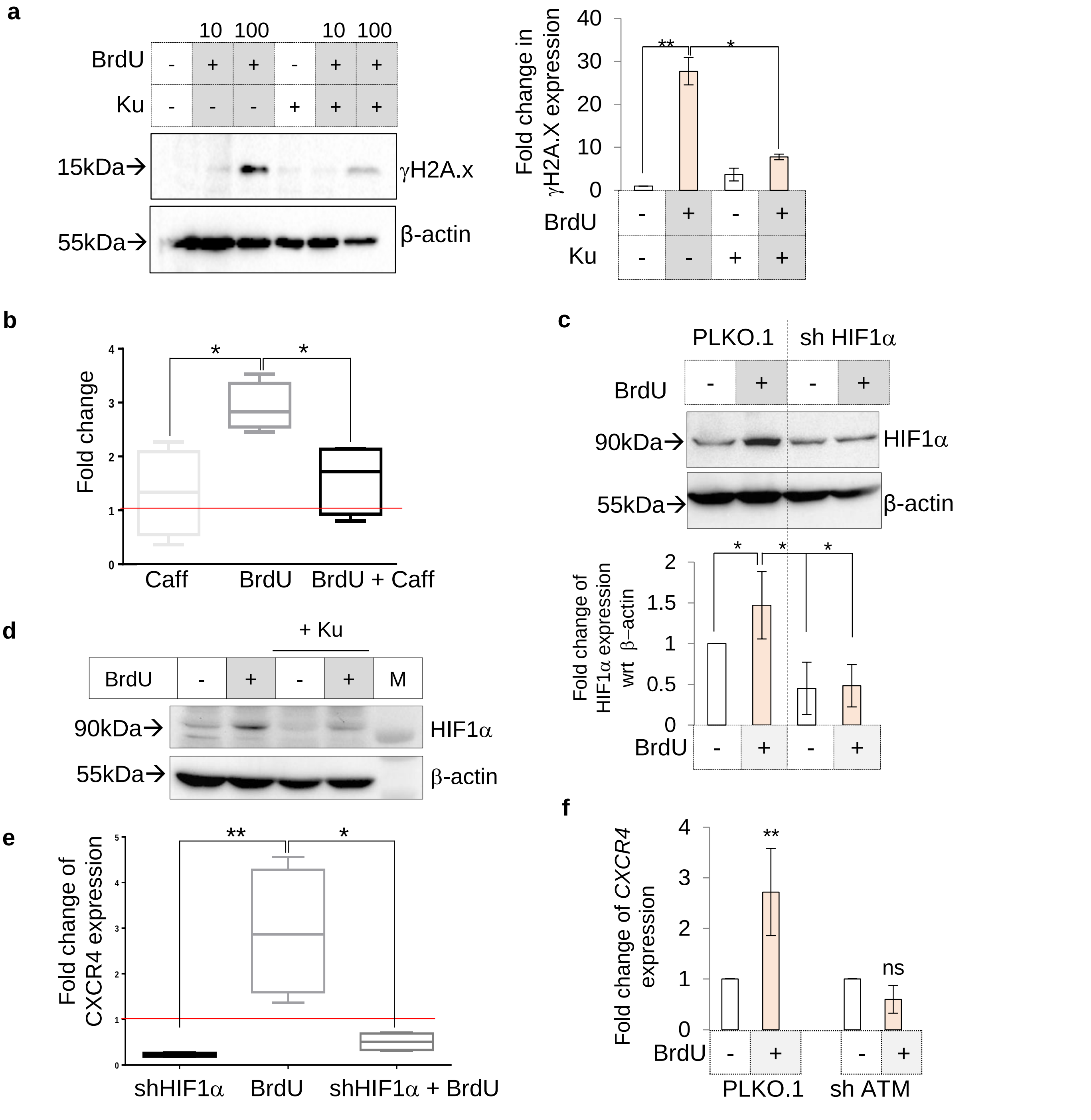

Fig. S2. Analysis of ATM kinase inhibition cellular senescence. (a) Analysis of effect of DDR. Lysates from HeLa cells treated for 48 hrs with Ku55933 alone and in presence of BrdU was evaluated for γH2A.x levels as a readout for ATM kinase activity. Quantification for blots is shown in the right panel. **(b) Analysis of CXCR4 expression by flow cytometry.** HeLa cells treated with BrdU in presence of absence of caffeine and CXCR4 surface expression was analysed by flow cytometry. Expression levels in control untreated cells was considered as 1 (marked by red line) for analysis. **(c) Analysis of HIF1α expression during DDR.** HeLa cells were treated with BrdU for 48 hrs and evaluated for HIF1α expression by western blotting. Also, shRNA was used to knockdown HIF1α expression in HeLa (n=3). Results are shown as mean ± s.e.m. * p value ≤ 0.05 (Student's t-test; n=3). **(d) Analysis of HIF1α expression by using western blotting.** Lysates from HeLa cells treated for 48 hrs with Ku alone or in presence of BrdU and evaluated for HIF1α levels. **(e) Expression analysis for CXCR4 receptor in HIF1α knocked down cells by flow cytometry.** The analysis and experiments were performed as per 2b. **(f) Analysis of CXCR4 expression in ATM kinase knockdown cells.** HeLa cells transfected with pLKO.1 vector or shRNA targeting ATM kinase were analysed for expression of *CXCR4* receptor before and after treatment with BrdU by qRT-PCR analysis. The values were normalized to *GAPDH* expression and then wrt control cells to calculate fold changes. Results are shown as mean ± s.e.m. * p value ≤ 0.05; ** p value ≤ 0.01 (Student's t-test; n=3)

Supplementary Fig 3.

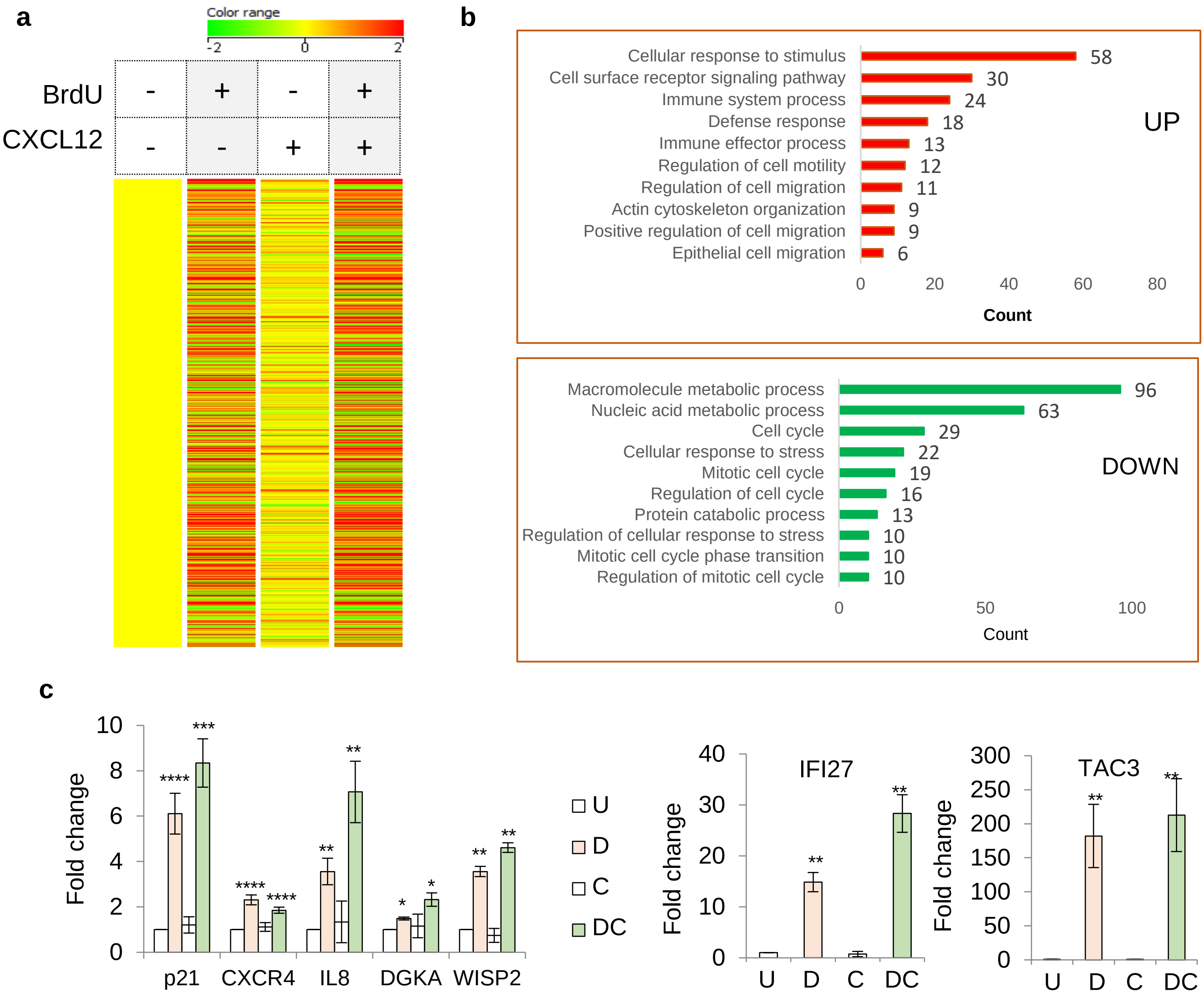

Fig. S3. Gene expression analysis to identify genes which are differentially regulated by activation of CXCR4 receptor during in presence or absence of DNA damage. (a) Heat map showing gene expression changes in cells treated with BrdU or CXCL12 and BrdU + CXCR12 as indicated. HeLa cells were treated as indicated for 48 hours and gene expression changes with respect to untreated control cells were analysed by microarray. Heat map was generated based on expression changes and the color range depicts fold change of genes expressed. (**b) Functional classification of genes with altered expression in presence of CXCL12 and DNA damage.** The genes, which were differentially regulated by CXCL12 during senescence induction but were not altered in presence of BrdU or CXCL12 alone were used for DAVID-gene ontology based analysis for biological processes. Gene families where genes were significant upregulated (top panel) and downregulated (lower panel) are shown. **(c) Validation of some of the differentially regulated expressed genes identified by the microarray analysis.** Changes in expression of *p21*, *CXCR4*, *IL8*, *DGKA*, *WISP2* (left), *IFI27* and *TAC3* (right) was examined in HeLa cells by qRT-PCR. Expression was normalized to *GAPDH* expression levels and then wrt to control untreated cells to determine the fold changes. (U, untreated; D, damaged cells; C, treated with CXCL12 and DC, damaged cells in presence of CXCL12. Results are shown as mean ± s.e.m. * p value ≤ 0.05; ** p value ≤ 0.01; *** p value ≤ 0.001; **** p value ≤ 0.001 (Student's t-test; n= or < 3). All p values are calculated wrt the untreated control cells.

Supplementary Fig 4.

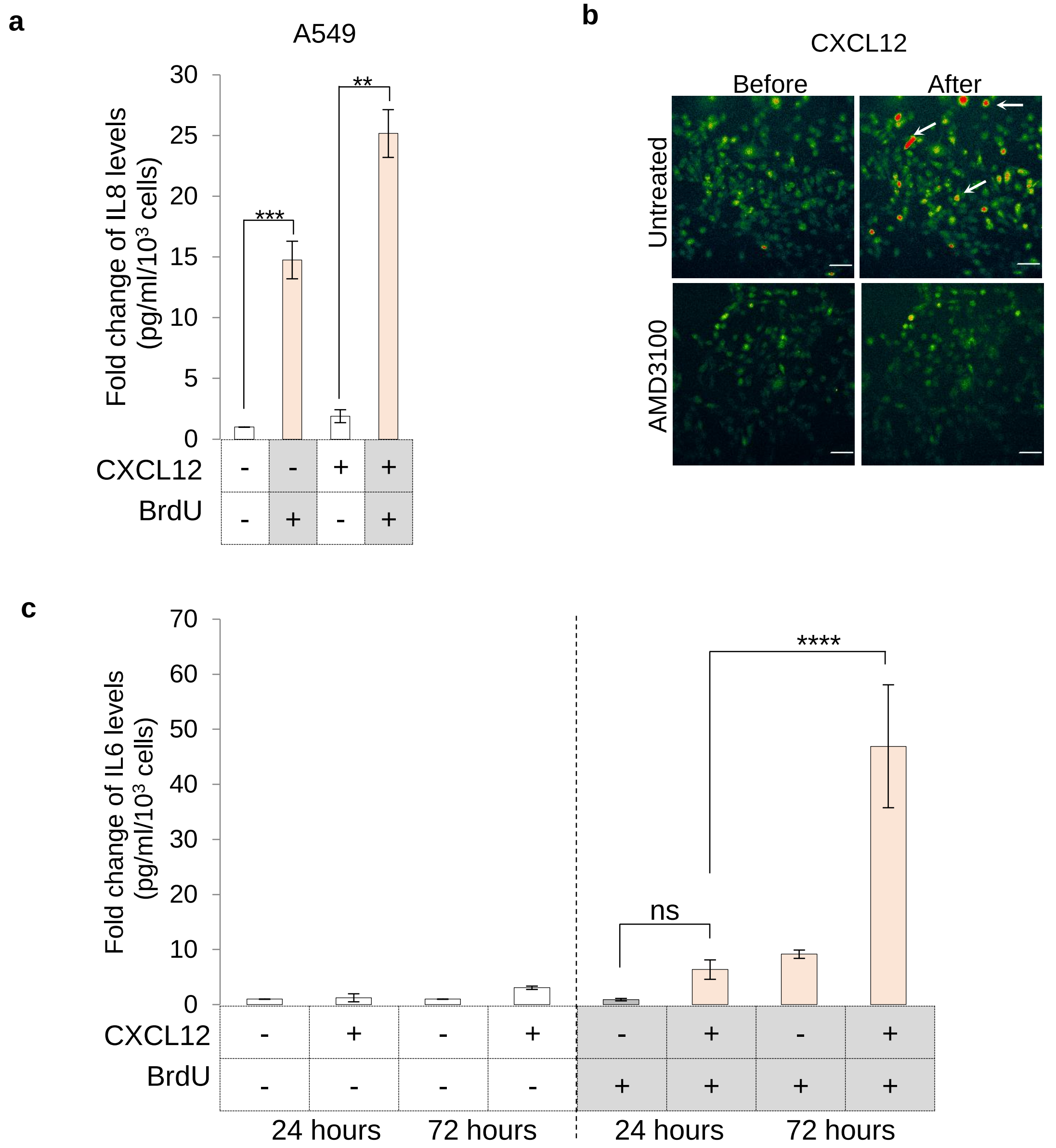

Fig. S4. Effect of GPCR signaling activation on DDR-dependent inflammation. (a) Measurement of IL8 **levels in response to CXCR4 receptor activation during DNA damage in A549 cells. (b) Calcium release assay to evaluate inhibitory effect of AMD3100 on CXCR4.** HeLa cells were lincubated with Fluo-4 dye for 20 minutes and imaged continuously, while stimulation with CXCL12 was done on untreated (above) and AMD3100 treated (below) cells. Image shown is before (left panel) and one second after CXCL12 treatment (right panel) from a time-lapse movie recorded under fluorescent microscope. Images are pseudocoloured were red colour indicates highest intensity. Scale bar = 50µm **(c) Measurement of IL6 levels in cells after CXCR4 activation during DNA damage.** The measurements were done at earlier time points (24 hours) when cells are not yet senescent and later time point (72 hours) when cells were senescent (n=3). For all measurements the media from treated HeLa cells (as indicated) was used for determining the fold change of indicated molecule secreted per 10^3 cells compared to untreated control cells. All results are mean ± s.e.m. p value * < 0.05, **** < 0.001 (Bonferroni's multiple comparisons test).

Supplementary Fig 5.

a

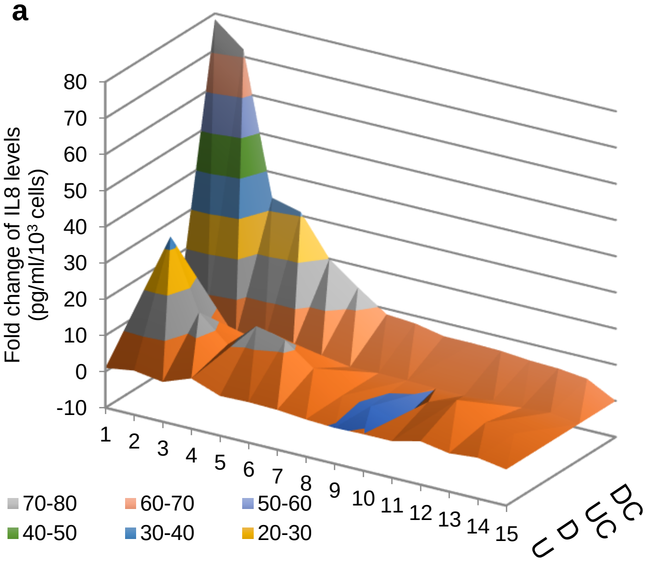

b

	Treatment / Compound	U	D	UC	DC
1	DMSO/Control	1.0	12.1	4.8	78.4
2	Ion Pump blocker	2.2	33.0	4.8	72.0
3	Sodium channel blocker	1.0	14.0	0.7	33.0
4	Potassium ion blocker	3.9	7.0	1.0	31.0
5	Glutamate antagonist	1.0	14.0	1.5	20.0
6	PDE4A inhibitor	1.2	12.0	1.5	14.0
7	Calcium Antagonist	1.0	6.2	0.3	8.5
8	8-Br-cAMP-Na Salt	0.3	0.6	0.5	7.9
9	Collagen synthesis inhibitor	-0.1	-0.4	0.4	6.3
10	Lipoxygenase inhibitor	0.1	-3.0	0.2	6.3
11	ACAT inhibitor	0.1	4.6	0.1	6.1
12	Chloride blocker	2.0	7.1	0.7	5.4
13	Reserpine	0.6	2.8	0.6	5.1
14	p38 MAPK inhibitor	1.3	1.7	1.2	4.2
15	Budesonide	0.0	0.0	0.0	0.0

c

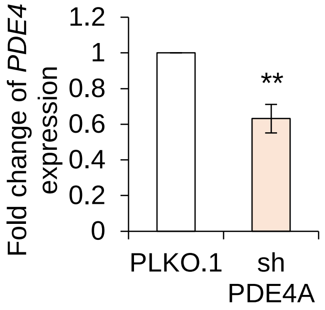

d

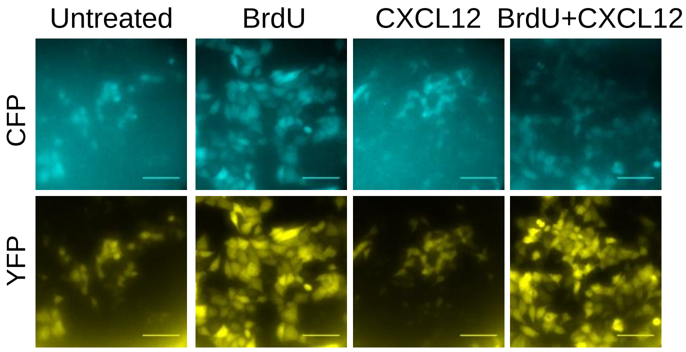

e

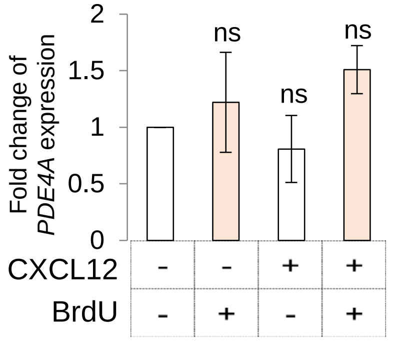

Fig. S5. Screening of LOPAC library to identify target molecules involved in CXCR4 receptor activation dependent inflammation enhancement during DNA damage. (a) A 3D contour plot to report the effect of compounds on inflammatory cytokine levels in various treatment conditions. The levels of IL8 were evaluated using the supernatant from treated cells (as indicated) as described in Materials and Methods. The plot shows IL8 levels, represented as fold change of IL8 (pg/ml) secreted per 10^3 cells on the Y axis and the X axis represents individual compounds and the Z-axis represents the treatment conditions evaluated in the screen. Compound alone (U), with damage through BrdU (D), with CXCL12 (UC) and with BrdU damage and CXCL12 together (DC). All values are with reference to level of IL8 recorded from cells treated with DMSO alone without DNA damage. (Sample 1 labelled on X axis is DMSO alone and 2-15 are compounds in the LOPAC which are listed in **(b)**. **(c) Analysis of *PDE4A* knockdown.** HeLa cells transfected with scrambled shRNA or one targeting *PDE4A* were analysed for expression changes by qRT-PCR. The values were normalized to *GAPDH* expression levels and pLKO.1 transfected cells to calculate fold changes. **(d) Analysis of cAMP levels using FRET based cAMP sensor, ICUE3.** Representative images captured in CFP and YFP channels for conditions (as indicated). Scale bar = 100 µm. Images were captured across different conditions with same acquisition settings. **(e) *PDE4A* expression analysis by qRT-PCR.** HeLa cells were treated with BrdU, CXCL12 or both for 48 hours and analyzed for *PDE4A* expression. The values were normalized to *β-actin* expression levels and compared to control to calculate fold changes. P values were calculated in reference to untreated and unstimulated cells.

Supplementary Fig 6.

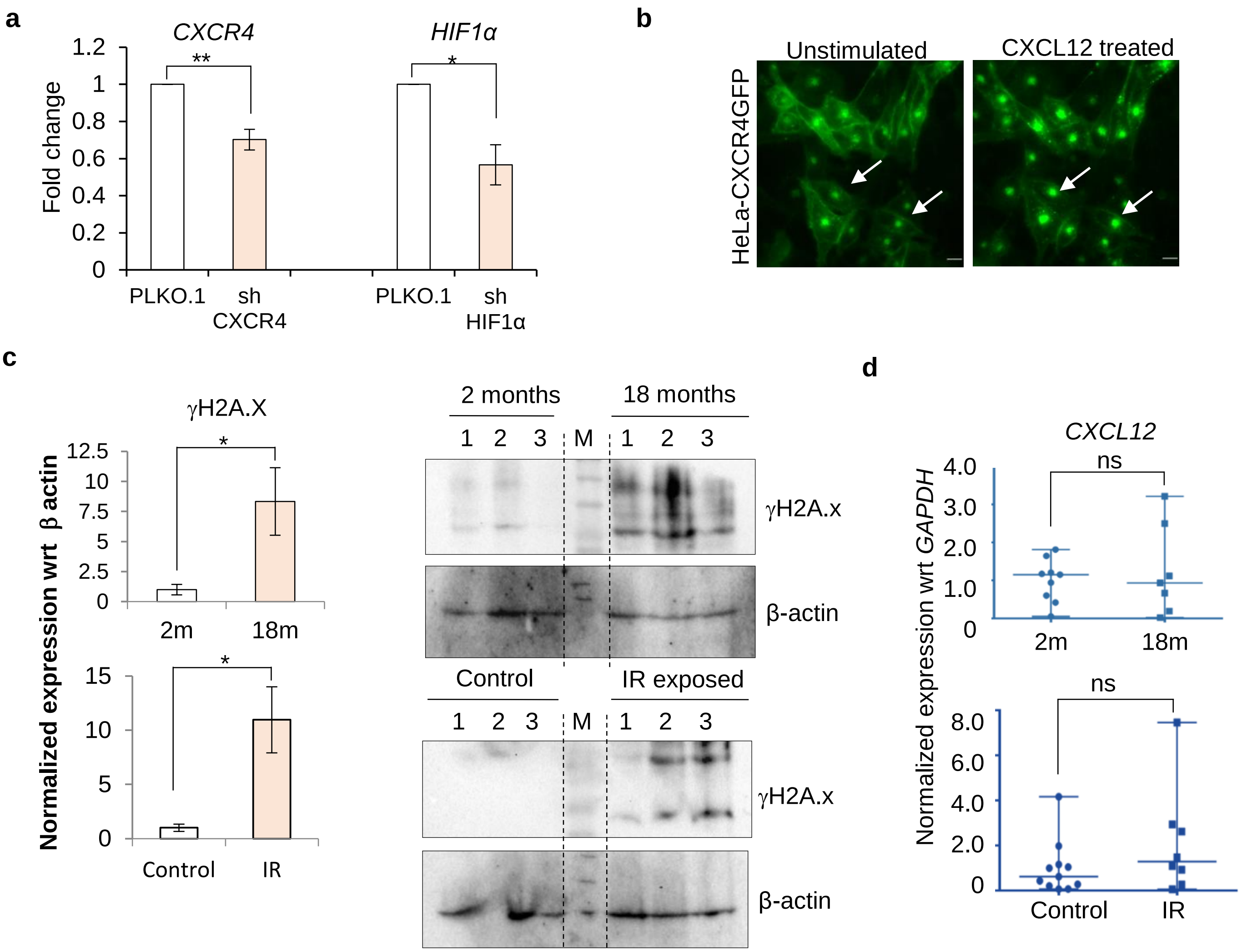

Fig. S6. Analyses of CXCR4 receptor expression and activation in cells and animals. (a) Analysis of expression levels of CXCR4 and HIF1α in shRNA mediated knockdown cells. HeLa cells transfected with pLKO.1 or with shRNA targeting *CXCR4* or *HIF1α* were analysed for gene expression changes using qRT-PCR. The values were normalized to *GAPDH* expression levels and pLKO.1 transfected cells to calculate fold changes. **(b) Characterization of CXCR4-GFP overexpressing HeLa cells.** Cells transfected with CXCR4-GFP plasmid were imaged before and after stimulation with its ligand CXCL12. Left, unstimulated (before) and right, stimulated (after) (scale bar = 10 μm). The images were taken over time from same dish and the internalized receptor is marked with arrows. **(c) γH2A.X analysis for presence of DDR in mice.** Both naturally ageing (top) and IR induced (bottom) mice show DDR when analyzed for γH2A.x through western blotting from liver tissue (left) and levels were compared by densitometric analysis (right) (n=3). **(d) *CXCL12* expression levels during aging in mice.** *CXCL12* expression was evaluated in liver tissue from naturally aged mice (top) or IR treated mice (bottom) compared with controls by qRT-PCR.

Supplementary Fig. 7

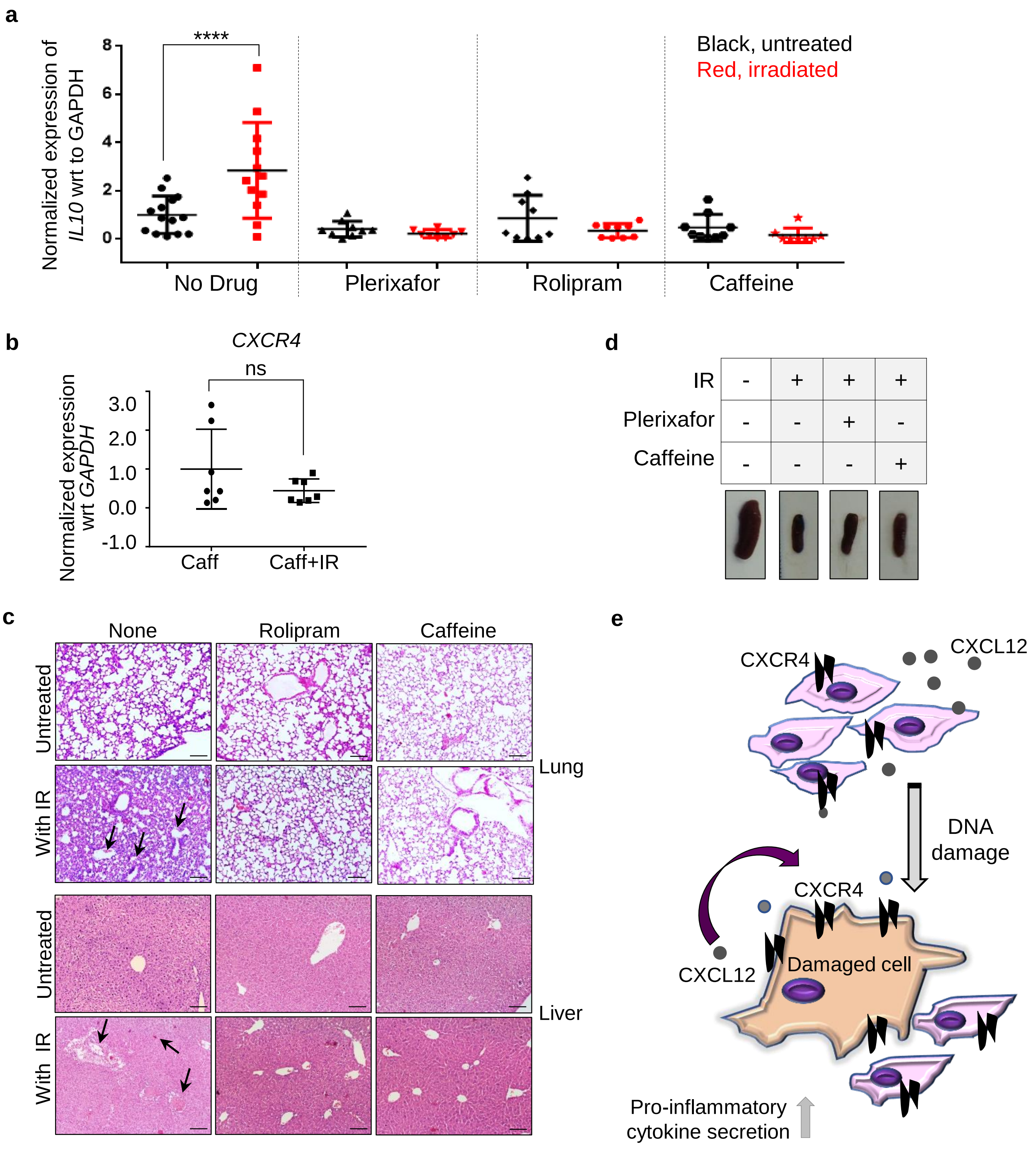

Fig. S7. *In vivo* analysis of effect of CXCR4 signaling during DNA damage in mice. (a) *IL10* analysis in mice tissue. qRT-PCR analysis for *IL10* in liver tissue of IR treated mice (red) and in presence of Caffiene, Rolipram and Plx. Black dots represent untreated and red dots represents irradiated mice. **(b) Analysis of *CXCR4* levels in presence of caffeine (ATM kinase inhibitor) in mice.** Mice pretreated with caffeine were irradiated and qRT-PCR analysis was done to measure changes in *CXCR4* levels in liver. **(c) Histopathological evaluation of liver and lung tissues in mice subjected to various drug treatments as indicated.** Animals were subjected to IR (6 Grays) and various treatments, sacrificed, and tissues were collected in 4% paraformaldehyde and processed for hematoxylin and eosin (H&E) staining as described in materials and methods section. The samples were imaged at 10x magnification (Scale=100 μm). **(d) Effect of IR treatment on spleen size.** Spleen from treated mice (as indicated) were surgically removed and photographed (n=7). **(e) Model for role of CXCL12-CXCR4 axis during DNA damage.** DNA damage triggers local induction of CXCR4 that causes enhanced CXCR4 signaling resulting in increased inflammatory-response.

Table S1. List of validated shRNAs used in the study.

Gene name	Accession number	ShRNA cat. no.	Sequence
CXCR4	NM_001008540.1, NM_003467.2	NM_003467.x-844s1c1	CCGGGCTGCCTTACTACATTGGGATCTCGAGATCCCAATGTAGTAAGGCAGCTTTTT
		NM_003467.x-226s1c1	CCGGCATCATCTTCTTAACTGGCATCTCGAGATGCCAGTTAAGAAGATGATGTTTTT
		NM_003467.x-346s1c1	CCGGCTTTGTCATCACGCTTCCCTTCTCGAGAAGGGAAGCGTGATGACAAAGTTTTT
		NM_003467.x-295s1c1	CCGGGAGAAGCATGACGGACAAGTACTCGAGTACTTGTCCGTCATGCTTCTCTTTTT
		NM_003467.x-1431s1c1	CCGGGAATCACGTAAAGCTAGAAATCTCGAGATTTCTAGCTTTACGTGATTCTTTTT
ATM	NM_000051.3,NM_138292.3	NM_000051.2-9426s1c1	CCGGCCTGCCAACATACTTTAAGTACTCGAGTACTTAAAGTATGTTGGCAGGTTTTTG
		NM_000051.2-224s1c1	CCGGGCCGTCAACTAGAACATGATACTCGAGTATCATGTTCTAGTTGACGGCTTTTTG
		NM_000051.2-1717s1c1	CCGGGCCATAATTCAGGGTAGTTTACTCGAGTAAACTACCCTGAATTATGGCTTTTTG
		NM_000051.2-7549s1c1	CCGGGCACTGAAAGAGGATCGTAAACTCGAGTTTACGATCCTCTTTCAGTGCTTTTTG
		NM_000051.2-9211s1c1	CCGGCGTGTCTTAATGAGACTACAACTCGAGTTGTAGTCTCATTAAGACACGTTTTTG
PDE4A	NM_001111307.1,NM_001111308.1,NM_001111309.1, NM_006202.2	NM_006202.1-3043s1c1	CCGGCCTTCCACTTTGGGTTCACTTCTCGAGAAGTGAACCCAAAGTGGAAGGTTTTTG
		NM_006202.1-612s1c1	CCGGGCATAGTAACAGCCTGAACAACTCGAGTTGTTCAGGCTGTTACTATGCTTTTTG
		NM_006202.1-1744s1c1	CCGGGAAATATCAATGGCCCAGATACTCGAGTATCTGGGCCATTGATATTTCTTTTTG
		NM_006202.1-437s1c1	CCGGCAGAGTACATTTCCACAACATCTCGAGATGTTGTGGAAATGTACTCTGTTTTTG
		NM_006202.1-1857s1c1	CCGGCCAGGAGTCGTTGGAAGTTATCTCGAGATAACTTCCAACGACTCCTGGTTTTTG
HIF1α	NM_001530.3,NM_181054.2	NM_001530.x-1492s1c1	CCGGCCGCTGGAGACACAATCATATCTCGAGATATGATTGTGTCTCCAGCGGTTTTT
		NM_001530.x-2671s1c1	CCGGCCAGTTATGATTGTGAAGTTACTCGAGTAACTTCACAATCATAACTGGTTTTT
		NM_001530.	CCGGGTGATGAAAGAATTACCGAATCTCGAGATT

		x-1048s1c1	CGGTAATTCTTTCATCACTTTTT
		NM_001530.x-369s1c1	CCGGCGGCGAAGTAAAGAATCTGAACTCGAGTTCAGATTCTTTACTTCGCCGTTTTT
		NM_001530.x-3867s1c1	CCGGTGCTCTTTGTGGTTGGATCTACTCGAGTAGATCCAACCACAAAGAGCATTTTT

Table S2. List of primers used in the study.

Primer Name	Primer sequence (5′ → 3′)
ACTB (β-actin)f	CCAACCGCGAGAAGATGAC
ACTB (β-actin)r	CAGAGGCGTACAGGGATAGC
*ATM*f	TTCAAAGGATTCATGGTCCAG
*ATM*r	GCTGTGAGAAAACCATGGAA
*CDKN1A (P21)*f	GGAAGACCATGTGGACCTGT
*CDKN1A (P21)*r	TAGGGCTTCCTCTTGGAGAA
*CXCL12*f	CGATTCTTCGAAAGCCATGT
*CXCL12*r	TCCAGGTACTCCTGAATCCACT
*CXCL8 (IL8)*f	GTGCAGTTTTGCCAAGGAGT
*CXCL8 (IL8)*r	CTCTGCACCCAGTTTTCCTT
*CXCR2*f	ACATGGGCAACAATACAGCA
*CXCR2*r	TGAGGACGACAGCAAAGATG
*CXCR4*f	CCGTGGCAAACTGGTACTTT
*CXCR4*r	TTTCAGCCAACAGCTTCCTT
CXCR7f	GGCTATGACACGCACTGCTA
CXCR7r	CTCATGCACGTGAGGAAGAA
P53f	AACAACACCAGCTCCTCTCC
P53r	CTCATTCAGCTCTCGGAACA
*DGKA*f	CCTTGAGGATCTTTGCCAGA
*DGKA*r	CAGTTTTGCCTCCTGTTGCT
*Gγ11*f	CGAAGATTTGCCAGAGAAGG
*Gγ11*r	AAGGGGTTCTTGTCTTCTGGA
GAPDHf	GAGTCAACGGATTTGGTCGT
GAPDHr	GACAAGCTTCCCGTTCTCAG
*HIF1α*f	GAAGTGGCAACTGATGAGCA
*HIF1α*r	GCGCGAACGACAAGAAA
*IFI27*f	CCACAACTCCTCCAATCACA
*IFI27*r	GCCTCTGCTCTCACCTCATC
*PCNA*f	AGGAGGAAGCTGTTACCATAGAG
*PCNA*r	AGGAGGAAGCTGTTACCATAGAG
*PDE4A*f	GTTCCTTCATCGTGGGTGAT
*PDE4A*r	AAGGATGTTGAACCGTGAGC
*TAC3*f	GAAAGGGTAACAGGAGCGTG

*TAC3*r	ATCCTCAAGTATCCCCCGAG
*WISP2*f	GGCCGTTCACCTCACAG
*WISP2*r	GCGACCAACTCCACGTCT
*mp21*f	CCTGGTGATGTCCGACCTG
*mp21*r	CCATGAGCGCATCGCAATC
*mCxcr4*f	GAAGTGGGGTCTGGAGACTAT
*mCxcr4*r	TTGCCGACTATGCCAGTCAAG
*mIL8*f	CAAGGCTGGTCCATGCTCC
*mIL8*r	TGCTATCACTTCCTTTCTGTTGC
mCxcl12f	GGTAGCTCAGGCTGACTGGT
mCxcl12r	TCCTCTTGCTGTCCAGCTCT
mActbf	GGCTGTATTCCCCTCCATCG
mActbr	CCAGTTGGTAACAATGCCATGT
mGapdhf	AGGTCGGTGTGAACGGATTTG
mGapdhr	TGTAGACCATGTAGTTGAGGTCA

Table S3. Subset of genes differentially regulated across BrdU and BrdU+CXCL12 treatments from the microarray analysis. The numbers indicate fold change in gene expression during BrdU or BrdU with CXCL12 treatments wrt Control (normalized as 1)

BrdU	BrdU + CXCL12	Gene Symbol	Related Gene family
4.14	4.83	CCL26	Chemokine
3.72	4.25	CCL28	Chemokine
3.47	6.43	CCL5	Chemokine
2.87	5.11	CCL20	Chemokine
2.1	4.97	CXCL10	Chemokine
2.85	3.38	CXCL14	Chemokine
1.00	1.81	CCL11	Chemokine
0.78	2.23	CCL25	Chemokine
0.84	1.73	CCL2	Chemokine
0.73	0.15	CCL27	Chemokine
1.34	4.00	CCR2	Chemokine
2.24	3.94	CCL15	Chemokine
2.25	2.97	IL8	Chemokine
1.79	0.82	CXCL11	Chemokine
1.86	2.42	STAT4	Chemokine
1.69	1.73	CXCR4	Chemokine
1.69	2.29	CCRL1	Chemokine
1.22	2.13	CXCL2	Chemokine
2.74	4.63	IL10	Cytokine
2.34	4.99	NOX5	Cytokine
1.40	2.46	TNF	Cytokine
1.32	0.61	IL16	Cytokine
1.31	2.54	KLF6	Cytokine
1.87	0.51	Sept 5	Cytokine
5.03	5.34	STAT3	Cytokine
1.39	4.15	MAF	Cytokine
2.85	1.30	TNFSF10	Cytokine
3.93	6.20	BMP15	Cytokine
2.64	3.04	IL26	Cytokine
1.31	2.54	KLF6	Cytokine
1.05	1.60	IL5	Cytokine

BrdU	BrdU + CXCL12	Gene Symbol	Related Gene family
3.03	4.46	IL6	Interleukin
2.34	4.99	IL32	Interleukin
2.25	2.97	IL8	Interleukin
3.07	5.22	IL7R	Interleukin
0.81	1.13	IL10	Interleukin
5.08	5.47	IL1RL1	Interleukin
3.25	2.13	CSF3	Interleukin
1.35	0.25	TOLLIP	Interleukin
4.49	5.22	CASP1	Interleukin
4.40	5.43	S100A9	Inflammation
2.88	4.66	CXCL11	Inflammation
1.41	7.79	PLA2G4C	Inflammation
2.96	4.59	NOS2A	Inflammation
1.79	0.82	CXCL11	Inflammation
5.87	5.99	AIF1	Inflammation
2.96	4.59	NOS2A	Inflammation
1.60	3.38	HRH1	Inflammation
1.34	3.00	CCR2	Inflammation
3.04	3.87	CDC14A	Cell Division
2.62	2.72	Sept 12	Cell Division
2.24	1.74	IGF2	Cell Division
1.87	0.51	Sept 6	Cell Division
1.76	2.46	CHFR	Cell Division
1.71	2.10	CDC2L6	Cell Division
1.20	1.16	CDC34	Cell Division
0.93	2.25	SPDYC	Cell Division
0.92	2.14	NEK3	Cell Division
4.44	5.10	CDKN1A	Cell cycle arrest
1.50	0.55	FOXO4	Cell cycle arrest
1.42	0.91	APC	Cell cycle arrest
2.23	2.07	CDKN2D	Cell cycle arrest
2.88	2.20	MLF1	Cell cycle arrest
2.55	1.95	CDC25A	Cell cycle arrest
3.72	5.16	EMP2	Proliferation
0.94	0.85	TGFB3	Proliferation
0.92	0.68	IL7	Proliferation
0.64	0.32	SP6	Proliferation
0.99	0.58	GDF11	Proliferation
0.56	0.78	CDK10	Proliferation

Table S4. Scoring for histology data.

Liver

Group	Animal	Canalicular architecture	Hemorrhage	Portal hyalinization
Untreated	1	fine	absent	absent
	2	fine	absent	absent
IR	1	impairment	present	present
	2	fine	present	absent
	3	fine	absent	present
Rolipram	1	fine	present	absent
	2	impairment	present	absent
	3	impairment	present	present
Rolipram + IR	1	fine	absent	absent
	2	fine	absent	absent
	3	fine	absent	absent
Caffiene	1	impairment	present	absent
	2	fine	absent	absent
	3	fine	present	absent
Caffiene + IR	1	fine	absent	absent
	2	fine	present	variable
Plerixafor	1	fine	absent	absent
	2	fine	absent	variable
Plerixafor + IR	1	fine	absent	absent
	2	fine	absent	absent

Lung

Group	Animal	Alveolar thickening	Hemorrhage	Fibroblastic foci
Untreated	1	Mild patchy atelectasis signs no thickening	No	No
	2	No	No	No
	3	No	No	No
IR	1	pervasive	present	Large
	2	patchy	present	small
	3	patchy	present	small
Rolipram	1	No	No	No
	2	No	No	No
Rolipram + IR	1	No	No	No
	2	small patches	No	No

	3	No	No	No
Caffiene	1	pervasive	present	large
	2	patchy	present	small
Caffiene + IR	1	patchy	present	large
	2	patchy with atelectasis	present	large
	3	No	No	small
	4	No (atelectasis)	No	large
Plerixafor	1	present	No	absent
	2	present	No	large
Plerixafor + IR	1	present with atelectasis	no	large
	2	present with atelectasis	no	small

Supplementary Methods

Cell culture

All cell lines used in the study were grown in DMEM (Sigma Aldrich) containing 10% FBS. The water was autoclaved before use followed by membrane filtration (0.2μm). Penicillin and streptomycin were always used in the culture media and in addition during early passage before experiments, cells were treated with plasmocin during passage 1 and 2, to eliminate mycoplasma contamination. Overall cell lines were used within 10 passages after thawing.

Microarray analysis

RNA Quality Control: The concentration and purity of the RNA extracted were evaluated using the Nanodrop Spectrophotometer (Thermo Scientific, USA; 1000). The integrity of the extracted RNA was analysed on the Bioanalyzer (Agilent; 2100).

Labeling: The samples for Gene expression were labeled using Agilent Quick-Amp labeling Kit (p/n5190-0442). 500ng each of total RNA were reverse transcribed at 40°C using oligo dT primer tagged to a T7 polymerase promoter and converted to double stranded cDNA. Synthesized double stranded cDNA were used as template for cRNA generation. cRNA was generated by *in vitro* transcription and the dye Cy3 CTP (Agilent) was incorporated during this step. The cDNA synthesis and *in vitro* transcription steps were carried out at 40°C. Labeled cRNA was cleaned up using Qiagen RNeasy columns (Qiagen, Germany) and quality assessed for yields and specific activity using the Nanodrop ND-1000.

Hybridization and Scanning: 600 ng of labeled cRNA sample were fragmented at 60ºC and hybridized on to an Agilent Custom Human Gene expression 8X60K Microarray designed by Genotypic Technology Pvt. Ltd. (AMADID: 027114). Fragmentation of labeled cRNA and hybridization were done using the Gene Expression Hybridization kit (Agilent Technologies). Hybridization was carried out in Agilent's Surehyb Chambers at 65ºC for 16 hours. The hybridized slides were washed using Agilent Gene Expression wash buffers and scanned using the Agilent Microarray Scanner (Agilent Technologies,).

Feature Extraction: Data extraction from microarray images was done using Agilent Feature Extraction software.

Gene expression Data Analysis: Feature extracted raw data was analysed using Agilent GeneSpring GX Version 12.1 Software. Normalization of the data was done using the 75th

percentile shift Significant genes up and down regulated showing one-fold (log base2) and above within the samples with respect to control sample were identified. Differentially regulated genes were clustered using hierarchical clustering based on Pearson coefficient correlation algorithm to identify significant gene expression patterns. Genes were classified based on functional category and pathways using Agilent GeneSpring GX and Genotypic Biointerpreter-Biological Analysis Software (Genotypic Technology Private Limited, Bangalore). Genes were also classified based on functional category and pathways using Biological Analysis tool DAVID (http://david.abcc.ncifcrf.gov/).

Western blot analysis. Cell lysate was prepared using ProteoJET Mammalian Cell Lysis Reagent (Fermentas Inc., USA) as per manufacturer's protocol. The total protein amount in the lysate was estimated using Bradford's protein estimation reagent. For analysis, 50 -100µg of total protein was resolved on 12.5% SDS-PAGE gel, transferred to PVDF membrane (GE Healthcare, USA) and protocol described previously was followed (Nair et al. 2014; Bagheri et al. 2017). All the primary antibodies were from CST (Cell Signaling Technology Inc., USA) and used at 1:1000 dilution, viz. phospho-histone γH2AX (Cat No. 9718), β-tubulin (HRP conjugate) (Cat No. 5346P), MAPK-CDK substrate (Cat No. 2325) and β-actin (HRP conjugate) (Cat No. 5125). The developed blots were imaged and analysed using ChemiDoc MP Imaging system (Bio-Rad Inc., USA) at multiple exposure settings. β-actin or β- tubulin expression was used as a loading control in all experiments.

SA-β gal staining for senescent cells. The protocol described by Dimri *et al.* was followed for SA βgal staining (Dimri et al. 1995). Cells were washed in PBS, fixed for 15 minutes at room temperature in 0.2% glutaraldehyde (Amresco, USA) in 1X PBS, washed three times with 1X PBS and incubated overnight at 37°C (without carbon dioxide) with freshly prepared SA βgal staining solution. The staining solution contains 1mg/ml of X-gal (GoldBio Technology, USA) in 40 mM citric acid/sodium phosphate, pH 6.0, 5mM potassium ferrocyanide, 5 mM potassium ferricyanide, 150 mM NaCl and 2 mM magnesium chloride (all chemicals from SRL, India). After overnight incubation, the cells were washed with 1X PBS and imaged with 20x objective for presence of blue colour in the cells. The imaging was done using an inverted IX81 microscope, equipped with DP72 colour CCD camera (Olympus, Japan).

Supplementary Figures
Unedited/ uncropped blots

Fig 3 (c)

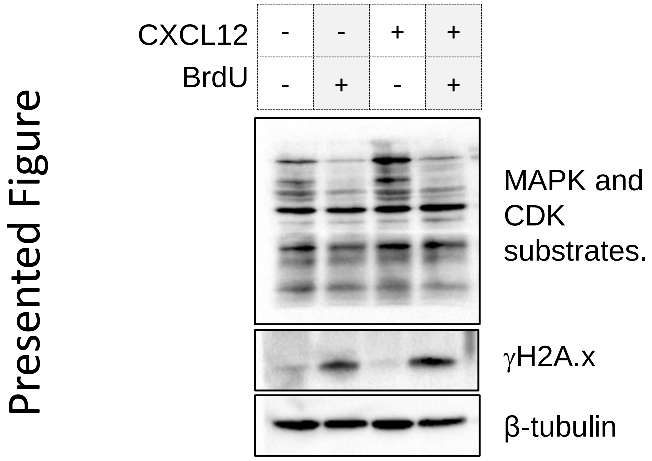

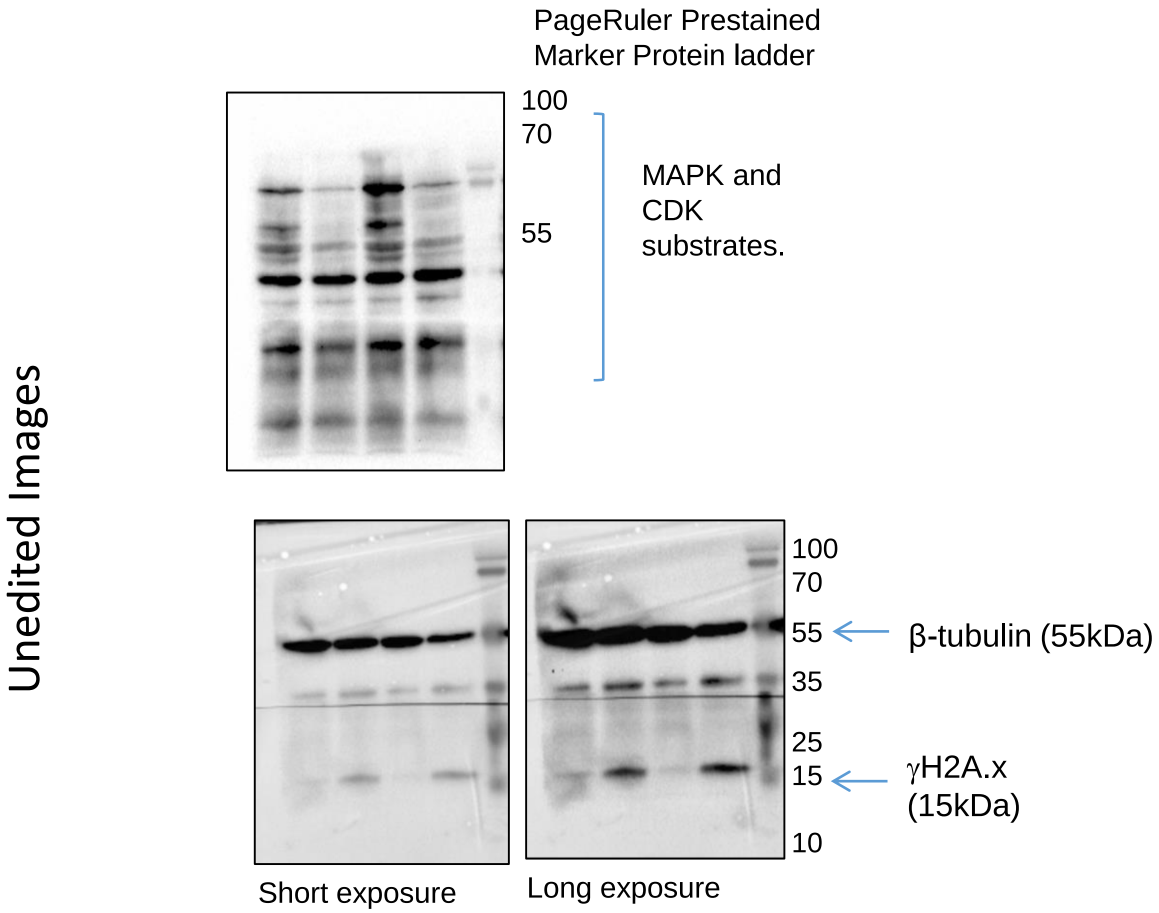

Supplementary Figure
Unedited/ uncropped blot

Suppl 2(a)

Presented Figure

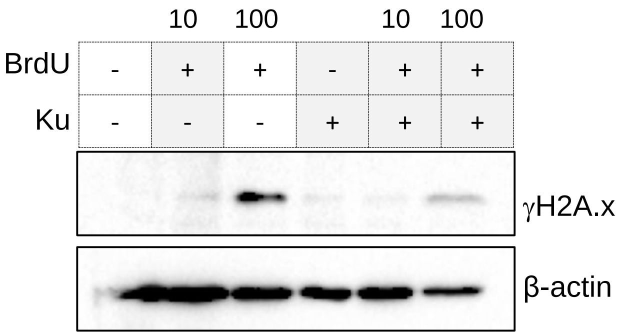

Unedited Images

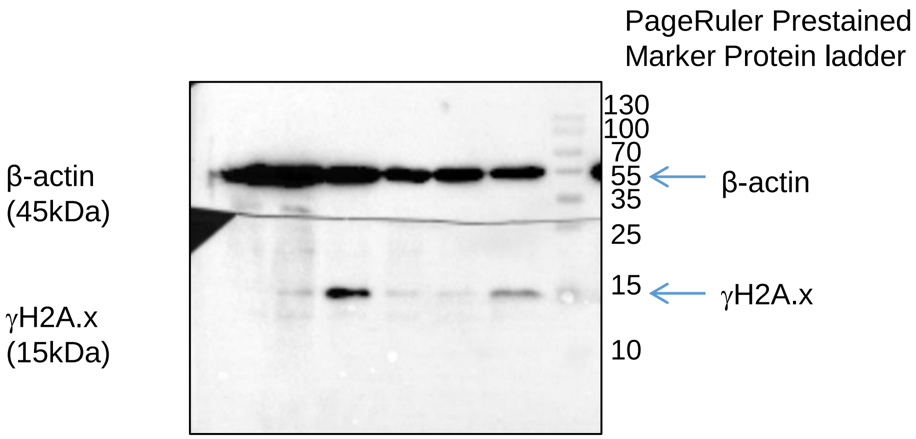

Supplementary Figure
Unedited/ uncropped blot

Presented Figure

Suppl 6(C)

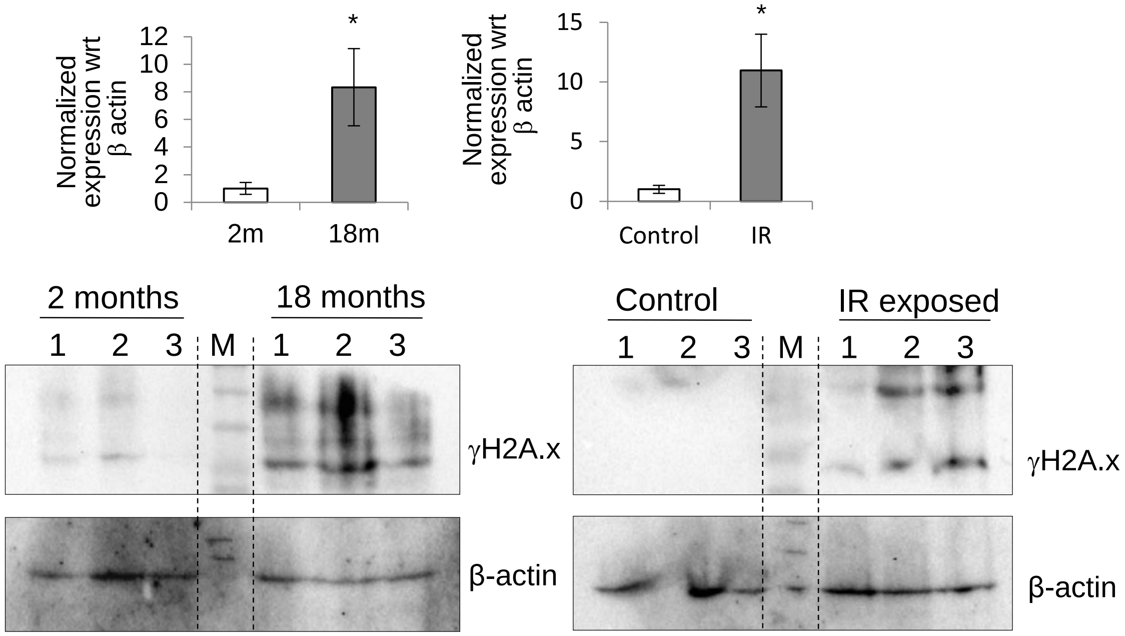

Unedited Images

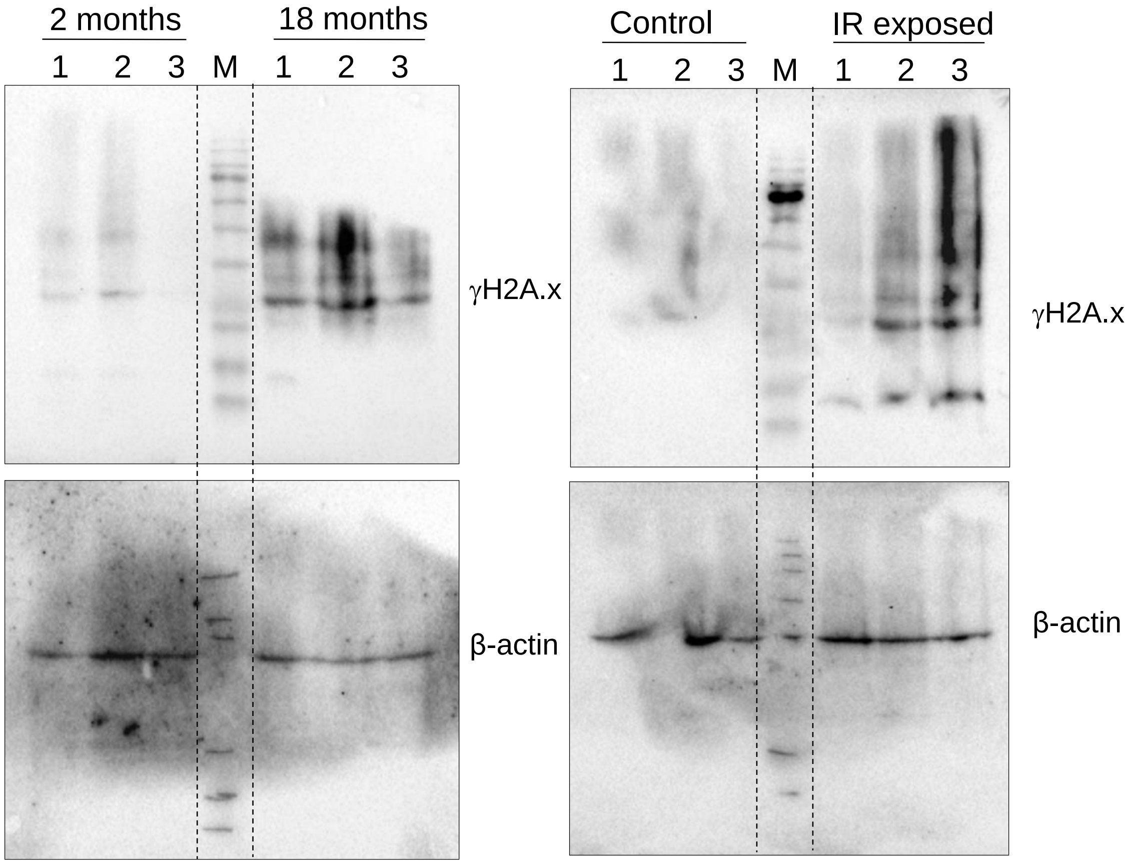

Supplementary Figure
Unedited/ uncropped blot

Suppl 2(d)

Presented Figure

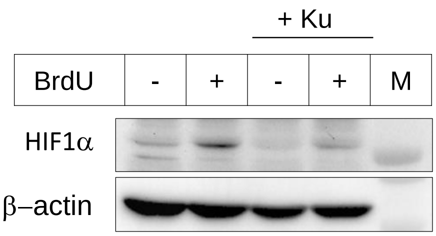

Unedited Images

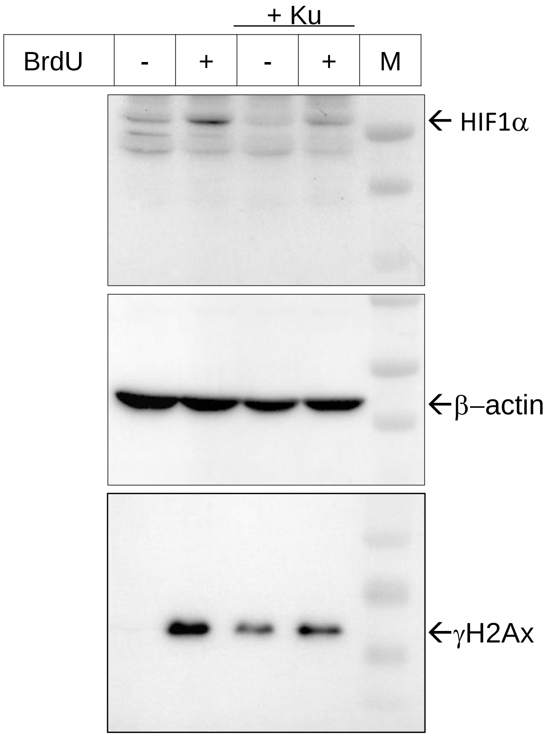

Supplementary Figure
Unedited/ uncropped blot

Presented Figure

Suppl 2(C)

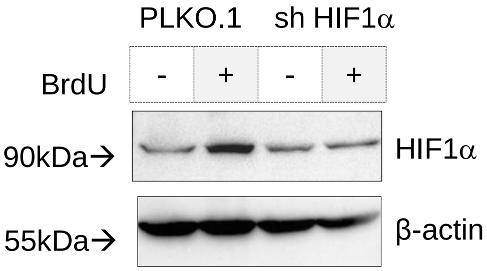

Unedited Images

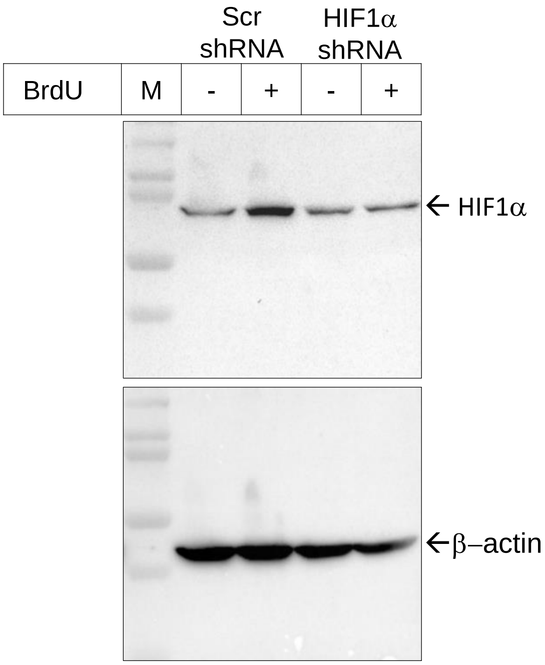

Supplement: Supplementary file 1 — SUPPLEMENTAL MATERIAL [file 41514_2018_28_MOESM1_ESM.pdf]
